# Broad Considerations Concerning Electrochemical Electrodes in Primarily Fluid Environments

**DOI:** 10.3390/ijms10052203

**Published:** 2009-05-18

**Authors:** Christopher G. Jesudason

**Affiliations:** Chemistry Department, Faculty of Science, University of Malaya, 50603 Kuala Lumpur, Malaysia; E-Mails: jesu@um.edu.my; chris_guna@yahoo.com; Tel. +603-7967-4270; Fax: +603-79674193

**Keywords:** capacitance, electrodes, Fermi surface, electronic chemical potential, electrochemical equilibria, Gouy-Chapman-Stern interface, potential of zero charge

## Abstract

This review is variously a presentation, reflection, synthesis and report with reference to more recent developments of an article – in a journal which has ceased publication – entitled “Some Electrode Theorems with Experimental Corroboration, Inclusive of the Ag/AgCl System” *Internet Journal of Chemistry*, (http://www.ijc.com), Special Issues: Vol. 2 Article 24 (1999). The results from new lemmas relating charge densities and capacitance in a metallic electrode in equilibrium with an ionic solution are used to explain the data and observed effects due to Esin, Markov, Grahame, Lang and Kohn. Size effects that vary the measured e.m.f. of electrodes due to changes in the electronic chemical potential are demonstrated in experiment and theory implying the need for standardization of electrodes with respect to geometry and size. The widely used Stern modification of the Gouy-Chapman theory is shown to be mostly inapplicable for many of the problems where it is employed. Practical consequences of the current development include the possibility of determining the elusive single-ion activity coefficients of solution ions directly from the expression given by a simplified capacitance theorem, the potential of zero charge and the determination of single ion concentrations of active species in the electrode reactions from cell e.m.f. measurements.

## Prologue

The review and report here describes work which had a particular philosophical approach. There were several levels or hierarchies that went into this study, following a pattern that others have pioneered. The study was predicated on attempting to delineate in a broad manner phenomena concerning electrodes that could be quantified to arbitrary degree. Such types of research begins at the qualitative level [1], leaving open areas for elaboration in the light of experimental and other theoretical developments. In this approach, the mathematics and concepts would have to be constructed for the problem at hand [2–4], and would appear elementary but not trite nor trivial, and lacking in the details and sophistication that is the result of syndicated activity with their high levels of coordination, organization and funding. Such alternative endeavors have been pursued by persons who for some reason or other [5] have been disengaged or detracted from the highly competitive and timely track of coordinated advance in the research field; this inadvertently allows such persons to explore areas that others would be reluctant to attempt by virtue of the set direction, momentum, competition and risk factors that accompany syndicated research activity. The work below – which appeared in *Internet Journal of Chemistry* as indicated in the abstract – did not and does not have the expected flavor of “timely results” that is a feature of the work output in syndicated and competitive fields of scientific research, but rather presents a basic examination of some fundamental assumptions – here in the field of electrochemistry – using the most elementary reasoning and techniques accessible to all, by first forming qualitative and elementary models. As a result, new results and ideas were formulated. In this inquiry, one notices that the ideas put forward are not motivated nor the result of competitively arriving at a solution first to a problem posed before a large contemporary academic audience, all encouraged to aim for the same prize as it were, often in a milieu that often presupposes the science itself to be a neo-Platonic race to adduce timeless and invariant truths which are independent of context, persons and situations. For an instance of non-timeliness, new interpretations are provided for the Esin and Markov effects and the observations due to Grahame, all of which were reported more than 60 years ago. Likewise, the new interpretations for all the other reported effects and their various assumptions which collectively have a history that extends nearly a century. The object therefore of this inquiry was not primarily to present results based on current trends generated by hot topics pursued by familiar teams and groups, but rather to illustrate that fundamental questions can be answered and theorems derived utilizing the most basic and most accessible of ideas that did not necessarily require privileged membership nor connections to influential research establishments, and this lack of large-scale support is in fact the familiar experience for the majority of enquirers in the peripheral regions of the globe, due to their not being historically nor culturally incorporated into such well organized and syndicated establishments. The broad scope of the analysis would therefore refer to work published over the century, some of which would seem quaintly dated, but nevertheless these references point to work which were all germinal in opening up new areas for consideration, and which left in their wake many unanswered questions. Further, the long time frames considered implies a rather long analysis, report and review that cannot be easily further abbreviated; fortunately, the advent of digital media and the internet has provided opportunities for extended treatments that would have been prohibitively costly using the more traditional technologies and media of just a few years back. The elementary forms proposed here are amenable to further refinement, modification and elaboration to any arbitrary degree, and therefore also provides a background for research projects that can be pursued further, thereby enriching the scope of research possibilities. And these pursuits need not be costly in terms of financial outlay, which is another serious bottleneck in most of Afro-Asia; the costs refer to the imponderables of personal labor, which is one affordable quantity found in many deprived environments. However, avenues for expression, support and value attribution are required to preserve the available commodity of personal labor and to prevent further attrition of the organizational and memory capacity that accompanies all learning experiences. The Epilogue provides a (brief) synopsis of some contemporary developments germane to the work. I would like to thank the Editor-In-Chief of *Internet Journal of Chemistry*, Steven Bachrach (Chemistry Department, Trinity University, San Antonio, Texas, USA) for permission to reproduce any figures, tables and images from the above mentioned article *(IJC*, Vol. 2, article 24) that appear here.

## 1. Introduction

Some of the key theories constructed to explain electrochemical phenomena seem to conform (understandably enough) to a linear superposition of independent physical concepts as a first approximation, without taking into consideration the possible special interference effects which might arise by juxtaposition of these concepts. An example of this approach is to be found in the general Stern modification of the basic Gouy-Chapman (GC) double layer theory. Here we observe a combination of the GC theory with the Helmholtz capacitance model, strictly without any interference. When interference effects are included, such as is suggested here (based on experimental and theoretical considerations) for the case of electrodes exchanging charge between the substrate electrode and the electrolyte across the Helmholtz inner layer, then the usual application of the Stern model to the electrode interface may not yield results compatible with experiment. The inadequacy here has nothing to do with the creation of “bad” theories or incomplete theories as opposed to better ones, nor the utilization of wrong concepts, but rather on not accounting for possible interference effects which would not be anticipated if the various key concepts were separately combined. Most of these cross-effects are of minor importance (such as those concerned with the electronic chemical potential), but others, such as those concerned with the double layer, can be quite dramatic, such as illustrated in Figure 10. Most of the theorems derived here preserve the sound and well established forms and structures of past workers, whilst taking into consideration the various interference effects which were neglected. All of these theorems are applied to systems already considered by others, and the data reinterpreted in the light of these new postulates, except for one relatively minor experiment, which was performed to test out ideas connected with i) a predicted e.m.f. difference due to variations in the chemical potentials, and ii) a new form of the chemical potential.

An example of minor interference is found in treatments of the solid state electronic chemical potential μ. In most electrochemical applications, it is inferred that since μ is an intensive quantity, it must be invariant with respect to system size, and since the electronic (or charge contribution) −Fψ_m_ accounts for the charge effects, it may be factored out of the pure, uncharged chemical potential contribution μ, which is in turn dependent on the free-electron (uncharged) number density ρ in the substrate lattice. On the other hand, there exists a charge transfer the instant the neutral electrode is inserted into the reactive electrolyte involved in the cell reaction, implying a change in the free-electron density ρ to ρ’. For fixed amount q of charge transferred, it is clear that ρ → ρ’ as the volume V of the substrate becomes large (V → ∞) since *q* /*V* → 0. This limit would correspond to the normal electrochemical assumption mentioned above. On the other hand, if the volume of the substrate electrode were to be diminished for fixed amount of charge q, then ρ’ → ∞ as V → 0, in principle. Since the experimental conditions lie somewhere in between these extremes, it would be reasonable to expect changes in the chemical potential of the electrode systems having charge transfer which would depend on their sizes relative to a standard reference electrode. In view of the tendency to miniaturize, especially where microelectrodes are concerned, it would follow those detectable and significant departures from the ‘ideal’ (i.e. V → ∞) cell e.m.f. would ensue as the electrode volume is reduced relative to a given standard electrode. As a minor portion of the present study, we include a low-resolution but unambiguous experiment to test the limits of validity of the common electrochemical assumption.

The fundamental premise in electrochemistry is that μ(e), the chemical potential of the electrons in the metallic phase substrate electrode is invariant during measurements [6–8] in systems at reversible equilibrium, and this μ(e) features in E^ø^, the standard potential of the electrode relative to some other electrode. E^ø^ values are determined [8] from the limit of values of the cell potential (by extrapolation) as the ionic concentrations tend to a specified value, and thus is a constant for the particular cell in question but it is an added assumption to suppose constancy of E^ø^ values when cells are concatenated [6] to determine activities for instance. If we should relax this constraint of constancy of μ(e) for the same concatenated cells, such as:

(1a) (s2) (−)Ag−AgCl |MCl aq. (m) | AgCl−Ag (+)(s1)

(with M = K^+^ for the system considered here), then the fundamental premise is contradicted if a nonzero and varying cell e.m.f. is registered for (1a) for different concentrations m. Furthermore, it would be beneficial if any of these cell potentials could be quantified by recourse to theory to map out the approximate regime of electrode size, solution strengths and the assumed potential of zero charge required in the theory where extraneous electrode effects become noticeable, so that in actual measurements, these predicted effects might be reduced or estimated by suitable choice of physical conditions.

In what follows, we use the conventional notion of electrochemical potentials with reference to an arbitrary spatial point for all components of interest [8] uncluttered by detailed refinements or comments [7]. The potentials mentioned, in particular, are for bulk properties, and this is the traditional understanding as well [6,8]. For instance, the strictly thermodynamical reaction equilibrium condition for the single electrode reaction:

(1b) $$\text{AgCl}(\text{s})+{\text{e}}^{-}(\text{Met}.)\;↔\;{\text{Cl}}^{-}\;(\text{aq})\;+\;\text{Ag}\;(\text{Met}.)$$

allows for a single electrode potential difference defined Δψ_d_ between the metal inner (bulk) Volta potential ψ_m_ and bulk solution ψ_s_ electrical potential (Δψ_d_ = ψ_m_ − ψ_s_) to be derived from equating the electrochemical potentials following the Gibbs equilibrium criterion:

(2a) $$\mu^{\text{O}}({\text{Cl}}^{-})\;+\;\mu \;(\text{Ag})\;+\;\text{RTln}\;\text{a}[{\text{Cl}}^{-}]\;-\text{F}\psi_{\text{s}}=\mu (\text{e},\;\text{Met})\;-\text{F}\psi_{\text{m}}+\mu \;(\text{AgCl})$$

Where:

(2b) $$\begin{array}{l} \Delta \psi_{\text{d}}\;=\;\psi_{\text{m}}-\;\psi_{\text{s}}\\ \;\;\;\;\;\;\;\;\;\;\;\;=\;\{-\mu (\text{Ag})-\;\mu^{\text{O}}({\text{Cl}}^{-}\;)\;+\;\mu (\text{e},\;\text{Met)}\;+\mu (\text{AgCl}\;)\}/\text{F}-(\text{RT}/\text{F})\text{lna}[{\text{Cl}}^{-}] \end{array}$$

with the standard potential E^ø^ identified as:

(2c) $$\{-\mu (\text{Ag})-\mu^{\text{O}}({\text{Cl}}^{-})\;\;+\mu (\text{e},\;\text{Met}.)\;+\mu (\text{AgCl})\}/\;\text{F}={\text{E}}^{\emptyset }$$

Henceforth, F is the Faraday constant, the μ′s are chemical potentials, T the Kelvin temperature, R the gas constant and the a’s represent activities. E^ø^ is set constant for activity coefficient determination at a fixed temperature. The electrochemical potential of the electron is defined as *μ̃*_e_ = *μ*(*e*, Met) − F*ψ_m_* has been factored out here to the bulk neutral metallic chemical potential μ(e, Met.) and that due to the metal Volta potential −Fψ_m_, and these are standard expressions [6–8]; Δψ_d_ is not the Volta potential but the equilibrium potential difference. We note that the Volta coupling potential between the electrode and external circuit are opposed at either ends of the circuit during actual e.m.f. measurements and their effects cancel; and hence for single-electrodes even, we may absorb the effects of the external coupling in say the μ(Ag) term. In particular, the experimental e.m.f. measurements of the electrochemical cell (1a) used to compute properties such as the activity coefficients are deemed to arise solely from the electrodes with no interference from the external circuitry, and the following analysis accords with this viewpoint in that the external circuitry effects are not considered. Equations (2) are (classically) exact equations irrespective of the potentials and concentration distribution between electrode, interface and solution for if we wrote down the equations coupling chemical potentials for each arbitrary region between the metal electrode and the bulk solution, then these equations would all cancel, yielding (2b) if the regions are in thermodynamical equilibrium, and so although the model used is connected to size and concentration changes across the interface, the computational results make use of the bulk concentration values or those derived from them (in the models for the AgCl dielectric layer) and therefore remain valid. In particular, perturbations in the measured values are due to variations in the terms of (2b). In (2), μ(e) is partitioned as μ(e, Met.) = μ_l_(e, Met.) + μ_F_(e) where μ_l_(e, Met.) is the energy (per mole) required to transport an electron to the metallic substrate lattice at the lowest energy where the electrons are counted and arranged according to Fermi statistics, and μ_F_ is the “Fermi energy” region chemical potential which can be defined precisely for certain situations, such as when the free electron gas model is utilized, where μ(e, Met.) is the chemical potential of the electron gas in a uniform, positively charged ‘jellium’ background (where the lattice contribution is ignored). This partition is standard for many experimental purposes, not least the rationalization of the T^3^ dependence of the electronic specific heat at very low temperatures. Here, the precision of the μ(e, Met.) difference determination used to measure electrode effects are dependent on the invariance of μ_l_(e, Met.), which implies non-distortion of the lattice; this can in principal be achieved by polarizing the electrode to the equilibrium value so that there is no change in lattice characteristics due to chemical (or metallic) deposition. The bulk potential ψ_m_ is linearly additive, i.e. *μ̃*(e) the electrochemical potential of the electron in the m metal substrate (we shall henceforth omit Met. which always signify the metal substrate) is always written as a linear decomposition of arbitrary terms, as long as these terms can be physically distinguished in principle, *viz*.

(2d) $$\tilde{\mu }(\text{e})=-\text{F}\psi_{\text{m}}+\mu_{1}(e)+\mu_{F}(e)$$

In order to test the regime of validity of E^ø^, the following lemmas (all of which have further applications to be elaborated) will be used. The results which the low resolution experiments obtained (despite the fact that these experiments could not determine unambiguously the predicted e.m.f. change of order of 0.1 mV with KCl concentration change of 0.1 m, compared to the actual cell e.m.f. values of ~ 1.5 mV over the entire concentration range) are in quantitative agreement to the theory developed from the lemmas. Each of the following lemmas will be applied to specific quantitative experiments. For what follows, the Greek letter symbols used follows the convention of the standard literature and this is sometimes unfortunate because of multiple usage of alphabets such as *ε*; the indicated subscripts or superscripts should leave unambiguous which quantity is implied.

## 2. The Theoretical Constructs

**Figure 1.**
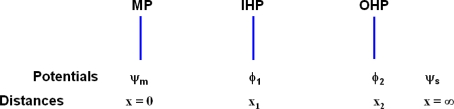
Schematic of the regions considered at the electrode interface.

In what follows, the different electrode types must be distinguished. The usual nomenclature [9] defines the: i) ideal polarized electrode, ii) ideal non-polarizable and iii) polarizable electrode; i) refers to an electrode which does not transfer charge between electrode and solution, and in particular the Gouy-Chapman (GC) and Stern modified (GCS) theories of electrodes [9] refer to such systems in the equilibrium (non-dynamical) net zero current regime. In the dynamical regime, when the potential relative to the bulk solution Δψ_d_ is varied, the current i must remain zero for this electrode (the i *vs* Δψ_d_ curve is horizontal). Thus the equilibrium situation of the inert electrode is extrapolated to the dynamical regime of a varying Δψ_d_ defined by (2) where no electric c urrent flows. By ‘inert’ is meant that there is no net exchange of ions or atoms (except electrons) between solution and electrode. The other extrapolation of behavior refers to ii) when the ‘equilibrium value’ Δψ_d_ (when i = 0) of the electrode system does not vary as a current is passed through the electrode-solution interface (the i vs Δψ_d_ curve is vertical) ; iii) refers to the intermediate situation, where the departure of the electrode potential from the Nernstian equilibrium value due to the passage of Faradaic current is a measure η of its polarization, written η = E− Δψ_d_′, where E is the measured electrode-solution potential for any current i and Δψ_d_′ the equilibrium e.m.f. relative to a reference electrode. We here also define the iv) self-polarizing electrode, which refers to an electrode which is its own source of the electrode-solution bulk e.m.f. Δψ_d_, without external impression. The many electrodes used in equilibrium electrochemistry is of this kind, when the electrode is reversible to one of the ions in solution, or provides a source for electron exchange, such as AgCl + e^−^(metal) → Ag + Cl^−^. The above definitions apply to what follows where only equilibrium steady state systems are considered. We also conform to standard usage of the definition of the inner and outer Helmholtz plane [9], (IHP and OHP respectively), where the IHP is the locus of the electrical centers of specifically adsorbed ions at distance x_1_ from the metal substrate plane (MP) and x_2_ is the nearest distance of approach for the solvated ions corresponding to the locus of centers for the OHP (where 0 < x_1_ < x_2_) and where the electric potentials at these points are ϕ_1_ and ϕ_2_ respectively. We note that for the GC and GCS models at *x =* ∞, ψ_s_ = 0, ∂ψ_s_/∂x = 0 to fit the boundary conditions. We define also ΔΨ_d_ = ϕ_2_ − ψ_s_, where ϕ_2_ → ψ_m_ as x_2_ → 0 in the absence of specific adsorption (when x_1_ = 0), and in this case ΔΨ_d_ → Δψ_d_. In this work both limits of x_2_ will be studied. The above setup is depicted in Figure 1, with the ϕ potentials associated with the various distances x.

### 2.1. Some Axioms and Results Applied To Electrodes

#### 2.1.1. Lemma (1): Concerning electrode charge

If there exists a function Q = Q(ΔΨ_d_, **n**), where Q is the surface charge density on the electrode and **n** are variables for the physical properties of the solution and electrode (such as the concentration of the electrolyte wherein the electrode is immersed), then since ΔΨ_d_ = ΔΨ_d_(Δψ_d_) is general and unspecified, it follows that if Δψ_d_ is the thermodynamically determined potential difference given by the Gibbs criterion (such as (2b)) then the potential ΔΨ_d_ gives the exact charge density on the electrode as Q(ΔΨ_d_) for fixed **n**. Hence, the two equations which are separate and independent may be coupled to yield one equation at equilibrium, viz. Q = Q(ΔΨ_d_). Under suitable conditions, one may (as discussed below) extend through superimposition the results of the inert electrodes of the GC and GCS theories pertaining to charge and potential distribution to that for self-polarizing electrodes provided the same mechanical structure is common to both. In particular if ΔΨ_d_ = Δψ_d_ (when x_2_ →0), the above formulation remains unaltered.

However, there is no proof that the converse follows, i.e. that from one coupled equation, the two separate equations may be recovered as is sometimes supposed[10]. An example of Q(ΔΨ_d_) is the simplified and well-known GCS Theory which gives:

(3a) $$\text{Q}(\Delta \Psi_{\text{d}},\text{n})={\left(8\text{kT}ɛ\;{ɛ}_{\text{o}}\;\text{n}\right)}^{1/2}\text{sinh}(\text{ze}\;\Delta \Psi_{\text{d}}/2\text{kT})$$

for a z-z electrolyte [9] by integrating Gauss’ theorem over the appropriate limits (x_2_ to ∞) where n is the electrolyte concentration, ε the permittivity, e the electronic charge, z the charge number, and k Boltzmann's constant [9] ; in particular ε, the permittivity refers to the bulk solution values. Further details of this electrode appear in (3b). Where there might be interference of other substances on the metallic substrate electrode such as AgCl in AgCl|Ag, we may apply a limiting process whereby the AgCl is (i) either placed at one portion of the electrode or else (ii) placed in a specular manner over the whole electrode surface with negligible surface area in both cases so that the interference effects are theoretically eliminated. On the other hand, we could include these effects by creating suitable theories, which will be attempted here. The above refer to the ideally polarizable GC model for charge density Q without specific adsorption for z-z electrolytes [9] which is equivalent to the expression for the GCS model (when ΔΨ_d_ = ϕ_2_ ≠Δψ_d_, and x_2_ finite and non-zero):

(3b) $$\text{Q}(\Delta \psi_{\text{d}},\text{n})={\left(8\text{kT}ɛ\;{ɛ}_{\text{o}}\;\text{n}\right)}^{1/2}\text{sinh}[\text{z}\;\text{e}/\text{2kT}(\;\Delta \psi_{\text{d}}-\text{Q}\;{\text{x}}_{2}/ɛ{ɛ}_{\text{O}})]$$

where x_2_ is closest distance approach to the surface for charges and are of magnitude for hydrated ions of ionic radii ~ 3 – 7 Å (for the ions of relevance here). Both these expressions are of course special case applications of the general expression (in the limit ∂ψ_s_/∂x = 0) for the electric field intensity dϕ/dx, which when integrated over the appropriate limits yields the charge via Gauss’ theorem, and is inferred by:

(3c) $${\left(\frac{d\phi }{dx}\right)}^{2}=\frac{2kT}{ɛ{ɛ}_{o}}\sum_{i}n_{i}^{o}\left[\text{exp}\left(\frac{-z_{i}e\phi }{kT}\right)-1\right]$$

for an ionic compound with species charged z_i_ with bulk concentration $n_{i}^{o}$. For single ion exchange, (eg. Cl^−^) as given in (1b), which we shall utilize, the charge on the metal substrate σ_m_ is given by solving the transcendental equation:

(3d) $$\sigma_{m}={\left(2kTɛ{ɛ}_{o}n_{o}\left[\text{exp}\frac{ze}{kT}\left(\Delta \psi_{d}-\frac{\sigma^{m}x_{2}}{ɛ{ɛ}_{o}}\right)\right]\right)}^{1/2}$$

In particular, the hydrated ionic radius for the chloride ion is 3.5 Å [11] and this value and others are used for x_2_ to demonstrate in Sec. III that for ‘dynamical’ interfaces, i.e. which is not ideally polarizable in that there is ionic exchange with the metal substrate, the model provided by Stern for ideal polarizability is simply not applicable, and direct experimental measurement also bears this out. The above lemma, together with Lemma 4 below was first derived and applied [12] to the Ag/AgCl system used to determine activity coefficients for aqueous chloride salts, where, if one of the electrodes was varied in size, it was proven that the derived activity coefficient was changed significantly, if the square silver substrate electrode was approximately below 25 μm in thickness (of length 0.707 cm). Recently, a replica of the above method, published first in reference [12] was applied (without the indispensable, detailed rationalization and discussion provided here for instance) for a problem in *irreversible thermodynamics* [13], where it was inferred that the Stern model of the interface gave the best results. Here, it is shown experimentally and computationally that for the strictly *equilibrium system* given by (1a), the Stern model surprisingly does *not* yield the experimental values unless x_2_ as defined above was very much less than 0.001 Å (~ 10^–4^ – 10^–7^ Å), which does not correspond to realistic ionic radii dimensions quoted in the studies. It is also obvious that the various GC and GCS models of electrodes were derived strictly for a system in thermodynamical equilibrium. It will be postulated that for the equilibrium case studies here, the Stern model is not applicable since the fixed x_2_ plane does not exist because any ion at the supposed closest distance approach (OHP at x_2_) may be absorbed via an electron transfer reaction that does not allow the construction of an average-distance x_2_ plane; the Stern model superimposes dynamical interactions of ions beyond x_2_ but fixes their position at averaged distance x_2_; for specific adsorption, x_1_ is also fixed but is not considered here (implying that the basic theories here may possibly be extended to incorporate these effects). In what follows the electrode used is not ideally polarizable (inert) but reversible to the specified ion (e.g. Cl^−^) because there exists an electronic pathway which exchanges the electrons on the substrate electrode with the chloride ions and AgCl, so that the invariant inner and outer Helmholtz planes of the Stern model (with no effective electronic charge distribution between them because of dynamical ion interactions) may be approximated as not existing, or its effects are relatively small.

#### 2.1.2. Lemma (2): Chemical Potential Considerations

For metals whose free electrons may be approximated by a highly degenerate electron gas, (and only a restricted set of metals may be so approximated) from (2c) we infer the introduction of electrons will only alter ψ_m_ and μ_F_(e) if the other lattice parameters are constant (so that μ_l_(e) is a constant).

We assume this to be the case since the volume change is theoretically never accounted for and is small compared to the bulk volume (V) of the electrode and from the statistical mechanics of the degenerate gas [14], the Fermi energy and chemical potential are unaffected by the gross potential of the electron gas. The electrode size parameter used here is the bulk volume V with assumption of no change in crystal structure and external pressure. As mentioned above, we may polarize the electrode before insertion to the solution to ensure that the lattice is not affected by deposition of neutral Ag atoms; thus all possible changes to the lattice core potential are eliminated. Under these conditions, the electronic properties can be treated separately from the invariant lattice parameters to first order, and this analysis is completely standard and in accordance with [14] the partition of the electronic chemical potential from the ion cores to determine properties due to electronic distribution [15]. The only parameters are the counting of the energy level relative to the energy of the lattice structure at the given electric potential of the metal electrode substrate. Hence, the change δν of the Fermi energy ν (where ν = ν_o_ + δν) is given for the highly degenerate electron gas without pairwise interactions by [14]:

(4) $$\text{v}=({\text{v}}_{\text{o}}+\delta \text{v})={\text{v}}_{\text{o}}{\left(1+\delta \text{N}/\text{N}\right)}^{2/3}$$

where ν_o_ = (h^2^/8m) (3N/πV)^2 / 3^ and δN is the mean number of electrons introduced from the neutral state. When ψ_m_ = 0, ν = ν_o_ and N is the electron number in the neutral state; m is the electronic mass and h the Planck constant. From the various expansion theorems [14], the chemical potential μ_F_ of the electron gas relative to the lattice potential energy μ_l_ is given up to fourth order by:

(5) $$\mu_{\text{F}}(\text{e})=\text{v}\;[1-\pi^{2}/12{\left(\text{kT}/\text{v}\right)}^{2}-\pi^{4}/80{\left(\text{kT}/\text{v}\right)}^{4}]$$

This expression, amongst others, will be used to compute μ_F_ which varies as charge is transferred to the electrode. The other definitions also used for computing μ_F_ will be given in the next section.

Clearly, not all metals conform to the above analysis, because of the Brilloun Zones formed by a periodic potential [16] which destroys the spherical symmetry of the momentum space energy distribution of the electrons. Ag, however is one exception, together with the Alkali and some other metals with regard to the experimentally measured thermodynamic properties derived from electron gas theory; the prediction of the low temperature specific heat for Ag from free electron gas theory (which sets μ_l_ = 0) accords extremely well with experiment, where the ratio γ_observed_/γ_theory_ = 1.0 (this agreeable ratio is unmatched by any other element in the periodic table) [17], with the electronic specific heat C expressed as:

(6) $$\text{C}/\text{T}=\gamma +{\text{AT}}^{2}$$

Other mechanical parameters cannot be directly correlated (such as the bulk modulus) since as noted μ_l_(e), the lattice contribution to the free energy becomes an important factor when compressing a solid, and with strong binding by lower-level electrons, the effects from μ_l_(e) will predominate over μ_e_(e). However, we are only interested in the thermodynamical properties when there is no distortion of the lattice. The thermal properties such as the observed and calculated Hall coefficients and Lorentz numbers are in close agreement[17] for Ag for this free electron gas model. A mapping of the Fermi surface [16,18] shows it to be spherical for Ag apart from apparent bulges in the <111> direction which is only of interest in the optical properties of the metal. As such, the assumption used here is that for mild perturbations of electron numbers, the free-electron Fermi sphere and its extensions to account for correlation effects is, from the experimental data, a very good approximation for the conduction band electronic distribution of Ag where thermodynamical properties are concerned.

#### 2.1.3. Lemma (3): Chemical Potential theorem

For the free electron gas jellium model, if the correlation and exchange energy is given (per particle) as ε_xc_, then the Fermi-Dirac density distribution [14] written *n̅* (*ε*) = [exp(*ε –μ*) / *kT +* 1]^–1^ (and which can be derived from the grand canonical ensemble) gives to first order the chemical potential as:

(7) $$\mu =\mu_{\text{F}}(\text{e})+{ɛ}_{\text{xc}}$$

with μ_F_(e) given by (5); n(ε) is the mean occupation number with energy ε, μ the chemical potential, k the Boltzmann factor and T the Kelvin temperature.

*Proof* : Since ε_xc_ is a total system property, then for each quantum number n within the Fermi sphere[14], the energy ε of that state is given by:

(8) $$ɛ=({\text{h}}^{2}/8{\text{mV}}^{2/3}){\text{n}}^{2}+{ɛ}_{\text{xc}}.$$

Consequently, the degeneracy g(ε) becomes:

(9) g(ɛ)=4π(ɛ−ɛxc)1/2(2m/h2)3/2Vdɛ  ;  ɛxc<ɛ<ɛo+ɛxc

where ε_o_ is the Fermi energy. By the substitution ε − ε_xc_ = ε′, the total number of particles N in the system becomes [14]:

(10) $$N=4\pi \int_{0}^{{ɛ}_{o}}{{ɛ}^{\prime }}^{1/2}V{\left(2m/h^{2}\right)}^{3/2}d{ɛ}^{\prime }\;\;;\;\;0\;<\;{ɛ}^{\prime }\;<{ɛ}_{o}.$$

Similarly, the Fermi-Dirac distribution transforms as:

(11) $$\bar{n}({ɛ}^{\prime })={\left[\text{exp}({ɛ}^{\prime }-\mu^{\prime })/kT+1\right]}^{-1}$$

and μ− ε_xc_ = μ′, 0 < ε′ < ε_o_.

Since [14]:

(12) $$N=\int_{0}^{\infty }\bar{n}({ɛ}^{\prime })g({ɛ}^{\prime })d{ɛ}^{\prime }$$

then (9), (10), (11) and (12) lead to:

(13) $$1=(3{ɛ}_{0}^{-3/2}/2)\int_{0}^{\infty }\bar{n}({ɛ}^{\prime }){{ɛ}^{\prime }}^{1/2}d{ɛ}^{\prime }$$

These expressions are now in standard form [14] for the typical expansion methods given for integrals of the form:

(14) $$J=\int_{0}^{\infty }\bar{n}({ɛ}^{\prime })X({ɛ}^{\prime })\;\;d{ɛ}^{\prime };\;\;X({ɛ}^{\prime })=\{\begin{array}{c} {{ɛ}^{\prime }}^{1/2}\\ {{ɛ}^{\prime }}^{3/2} \end{array}$$

The above expansion techniques lead to the standard series by elimination of μ′ as:

$$\mu^{\prime }={ɛ}_{o}\left[1-\frac{\pi^{2}}{12}{\left(\frac{kT}{{ɛ}_{o}}\right)}^{2}-\frac{\pi^{4}}{80}{\left(\frac{kT}{{ɛ}_{o}}\right)}^{4}+\ldots \right]=\mu_{\text{F}}(\text{e})$$

Hence:

(15) $$\mu =\mu_{\text{F}}(\text{e})+{ɛ}_{\text{xc}}$$

On the other hand [19] the theorem of Seitz claims to develop the chemical potential as ∂[n.ε_xc_]/∂n for the correlation and exchange part of the total energy (n being the density of the gas). This form can be traced to the use of the classical Gibbs type equation for intensive quantities by differentiation of the extensive free energy. The Fermi function and (15), on the other hand, is derived from the quantized grand canonical ensemble, subjected to the usual constraints of fixed particle number and energy, which utilizes the fundamental criterion of invariant chemical potential between any two members of the ensemble. It will be found that the experimental values of the cell potentials mentioned lie midway between those derived theoretically from the Seitz expression and the free electron model potential μ_F_(e). A hybrid form is used in computations [15] for the Seitz expression, where the total chemical potential μ_S_ is written as:

(16) $$\mu_{\text{S}}={ɛ}_{\text{o}}+\text{d}[\text{n}{ɛ}_{\text{xc}}(\text{n})]/\text{dn}$$

with ε_o_ obtained from the expansion techniques mentioned above (Equations14) whereas the other terms are derived by the thermodynamic analogue of the definition of the chemical potential μ (const. P,T), given by ∂E/∂N = μ(N) where E is the total energy, and N the particle number or concentration. Strictly, since E = 2Nε_o_/5 for the free electron gas for the non-interacting terms, the chemical potential for this system should be, according to the Seitz theorem ∂E/∂N ~ (3/5).ε_o_. The discrepancy sometimes found between results from numerical computations and experiments (e.g. the work function for the noble metals where a 30% discrepancy has been reported [15]) may be due in no small part to the chemical potential expression. We note that in the grand-canonical development of μ for a free electron gas, μ appears as a mere parameter [14] connected with the probability of the system having N particles where N varies. Thus no differential of the energy content is directly apparent from this definition of the chemical potential found in the Fermi-Dirac distribution.

For our computations, in addition to the form given by (5), which is in excellent agreement with the thermodynamical properties for Ag mentioned above, we will use two other expressions for the chemical potential for purposes of comparison, the often utilized [15] Seitz theorem μ_S_ (e) and the form derived from Lemma(3), μ_T_(e) which we write as:

(17) $$\mu_{\text{T}}(\text{e})=\mu_{\text{F}}(\text{e})+{ɛ}_{\text{xc}}.$$

The Seitz potential is [15]

(18) $$\mu_{\text{st}}(\text{e})=\mu_{\text{F}}(e)+\text{d}[\text{n}{ɛ}_{\text{xc}}(\text{n})]/\text{dn}$$

where n is the density of the electron gas. Wigner's interpolative expression, derived from quantum calculations for ε_xc_ is used above, where (in a.u.) [15]:

(19) $${ɛ}_{xc}(\bar{n})=-(0.458/r_{s})-0.44/(r_{s}+7.8)$$

with the electron density given by *n̄* = (N + δN)/V for any change of electron numbers δN on the electrode and r_s_ (in a.u.) is defined such that:

(20) $$4\pi r_{s}^{3}/3=1/a_{o}^{3}\bar{n}$$

where a_o_ is the Bohr radius (in S.I. units as with *n̄*).

#### 2.1.4. Lemma (4)

It is possible to determine the approximate value of the cell standard potential relative to the solution by electrocapillary maximum (e.c.m.) measurements (illustrated by the Ag/AgCl electrode).

The equilibrium single electrode Nernst equation relative to the solution ions is:

(21) $$\Delta \varphi_{\text{d}}={ℑ}_{\text{AgCl}|\text{Cl}}-\text{RT}/\text{Fln}[\text{m}.\gamma ]$$

for the Ag|AgCl electrode (with x_2_ = 0 or otherwise), and there is implied a value for ℑ_AgCl|Cl_ which must be estimated (henceforth ℑ^o^ = ℑ_AgCl|Cl_). At the electrocapillary maximum (e.c.m.), according to the normal interpretation of the Lippman equation [20,21] the charge on the electrode is zero, and thus, from the point of view of both the simplified Gouy-Chapman theory and the Stern modification (3a-d), the bulk potential between the metal electrode and solution must be zero; since at especially low concentrations of the active ionic electrode species, the relative surface excesses are practically equal to the surface excess concentrations [19]. Further, if it is argued that certain dipole arrangements preclude the normally interpreted zero potential difference, then it must be pointed out that this situation is not distinguishable from a charged ideally polarizable electrode with its separation of charge, which by definition constitutes a dipolar arrangement, where the Lippman equation is equally applicable. Hence at the point of zero excess charge, ℑ_AgCl|Cl_ may be determined because Δφ_d_ and the bulk activities of the ionic species are known in (21). This method was first applied to determine activity coefficient changes in a system whose two electrodes where varied in size [12], and recently a replica of this method was applied (somewhat nonchalantly, without the indispensable detailed justification) to the non-equilibrium electrokinetic problem [13] utilizing the equilibrium Stern electrode equation (3b).

#### 2.1.5. Lemma (5): A Simplified Capacitance Theorem

The term capacitance has undergone development in meaning ever since the solution of Laplace’s equation ∇^2^φ = 0 for a source free region with electrical potential φ. For a system of charges Q_i_ (i = 1, 2,…. N) in a region of a fixed relative permittivity ε_o_, Laplace’s equation [22] yields the solution:

(22) $$\varphi_{i}=\sum_{j=1}^{N}p_{ij}Q_{j}$$

for the potential at charged surface i, which on inversion is written:

(23,24) $$Q_{i}=\sum_{j=1}^{N}c_{ij}\varphi_{j};\;\;c_{ij}=\partial Q_{i}/\partial \varphi_{j}$$

is the coefficient of inductance (i ≠ j) and c_ii_ are called coefficients of capacitance, where these coefficients are all constant for a fixed geometrical configuration of charges; these expression are based on the early experimental results with conductors which showed remarkable linearity with regard to charge and potential variation within the available regime of variable values. In electrochemistry, the capacitance C_el_ of the electrode of charge Q with defined potential φ (relative to the bulk solution) is defined C_el_ = ∂Q/∂φ. Within the GCS theory the permittivity factor ε_o_’ (where ε_o_’ = ε.ε_o_ in 3(a–d)) is constant and C_el_ is not, implying that for any fixed number of ionic charges, the geometrical arrangement of these charges vary, and so the solution charge density ρ [9], which is given by the potential φ indirectly via Poisson’s expression ∇^2^φ = –ρ/ε_o_’, and φ(x) in each case varies according to the boundary conditions of the externally “impressed” e.m.f. at φ(0). In normal electrical (engineering) applications, variation in the capacitance is due to the variation in the dielectric coefficient for a fixed geometry, whereas here the mechanical structure (geometry) is the cause of the change; the electrochemical definition of the differential capacitance may be interpreted as a shorthand for describing systems that show different geometrical structures at different potentials and concentrations; in order to retrieve the charge on the electrodes, the appropriate integrations must be performed [9]. In particular, all quantities of physical interest, are linked to the potential φ distribution along the solution and interface (e.g. the surface charge density q is given by:

(25) $$q_{|x\prime }=ɛ.{ɛ}_{o}A(d\varphi /dx_{|x^{\prime }})$$

where these quantities are derived by appropriate differentiation and physical laws, such as Gauss’ law in electromagnetism.

The important question is whether in a self-polarizing electrode (where it is assumed that it is mechanically similar to the GCS models, except that in the GCS models the potential is externally impressed and may be varied) it is meaningful to define or deduce properties such as its ‘differential capacitance’ at its equilibrium potential, since it must be conceded that these potentials are invariant at any particular concentration of electrolyte. In what follows, it is proved that there is a meaningful definition of differential capacitance that is compatible with experiment provided the self-polarizing electrode is mechanically equivalent to a specified ideally polarizable (GCS usually but not necessarily) type. By ‘mechanical equivalence’ is meant that the ionic interactions are the same, and that the metal substrate of the electrode is also the same in all respects for the two electrode systems. With these assumptions, we model the ions in solution as minute “conductors” of arbitrarily small spherical radius δr_1_ and δr_2_ (δr_1_, δr_2_ → 0) for cation and anion respectively having a fixed quantum (±e) of charge, which implies the normal surface field strength:

(26) $$E_{i,n}=\partial \varphi_{i}/\partial n=\;\pm \sigma_{i}/{{ɛ}_{o}}^{\prime }$$

is fixed (*ε_o_′* is invariant throughout for the GCS theories) because of the fixed charge for each case of i = 1, 2 (cation and anion respectively). Even if these charges are not considered “conductors”, the above obtains for different values of *E’_i,n_* compared to *E_i,n_*. The bounding surface ∂V of this system is the electrode surface at x = 0 and the solution plane perpendicular to the x axis at x = l at arbitrarily large x (x → ∞), within which the conductor ions are suspended in a particular geometrical distribution consonant with thermal equilibrium and their surfaces are labeled S_j_ (j = 1, 2,... N) where N is fixed, large but finite, equal to the number of ions in the system; clearly these S_j_ are the internal surface boundaries of the system. The boundary conditions for the potentials are specified as φ_l_ = 0 for x = l, φ_o_ at x = 0 and as normal gradients ∂φ_j_/∂n at S_j_ (either φ or ∂φ_j_/∂n may be specified for each boundary at i).

Then, subject to these boundary conditions, the Uniqueness Theorem [22] states that *any two solutions φ(x) and φ‘(x) differ at most by an additive constant*, and since we specify *the potentials φ_o_* *and φ*_l_, the solution is unique to an additive constant relative to the postulated thermal averaged stationary distribution of charge at the specified temperature. Hence we reach the (remarkable) conclusion that for these electrodes, under appropriate conditions (such as fixed N, similarity of mechanical and electrical properties of the ions, and constant *ε_o_′*) there is one and only one potential function consonant with the physical properties of the system. Here, it is assumed that there is a unique thermally averaged (stationary) distribution of charges for any impressed e.m.f. for the GCS system. Clearly, the above solution to Laplace’s equation *does not distinguish externally impressed and locally impressed potential boundary conditions and hence we conclude that the self-polarizing electrode must have the same potential and solution charge distribution for the same mechanical and electrical properties of the ions* given *by such variables as φ_o_, φ_l_, N, ε_o_′ at any fixed temperature.* Since the surface charge density Q of the electrode is deduced via Gauss’ theorem, it is wholly dependent on the potential, i.e. Q = Q[φ], a functional of φ for the ideal polarizable electrode. Thus, under the assumption that the same mechanical structure exists for both the self-polarizing and ideally polarizable electrode, exactly the same surface charge density function Q’ exists for the self-polarizing electrode, i.e. Q’[φ] = Q[φ].

We infer that:

(27,28) $$\begin{array}{l} {\text{C}}_{{\text{d}}_{\text{x}}}=\partial Q/\partial \Delta \varphi ={\text{C}}_{{{\text{d}}^{\prime }}_{\text{x}}}\\ =\partial Q^{\prime }/\partial \Delta \varphi ,\;\text{where}\;\Delta \varphi =\varphi_{\text{x}}-\varphi_{o} \end{array}$$

for any potential *φ*_x_ at distance x from the electrode surface under the above assumptions; hence the differential capacitance (when x = 0) of the two systems are the same if *φ*_o_ and *φ*_l_ are specified. Thus for self-polarizing electrodes, we may by inference from solutions of Laplace’s equation still define the differential capacitance for the self-polarizing electrode as for (inert) ideally polarizable ones under the assumption of physical equivalence of these two electrodes and all physical properties may thus be derived from the appropriate mathematical operation. However, there is a difference; under the assumption of ideal polarizability, one can experimentally measure the differential capacitance directly for instance, whereas for the self-polarizing electrode, one must specify the mechanical properties by specifying the Q charge density function that the electrode is assumed to possess on physical grounds.

With the above understanding, we can state a simplified capacitance theorem for self-polarizing electrodes that for electrodes reversible to the ion(s) in the solution, the differential capacitance is independent of the equilibrium potential but is dependent on the physical characteristic variables **c** (such as the activity of the ion concerned) of the reversible electrode.

*Proof*: Suppose that the charge on the electrode is given by Q = Q(Δφ, **n**) where **n** are the physical variables pertaining to the electrode (permittivity, temperature etc.) whilst Δφ is the bulk interfacial potential written Δφ = φ_o_–φ_l_. Then dQ/dΔφ = Q′ (Δφ, **n**) = C_d_, the differential capacitance. But Δφ = Δφ (**c**) for this electrode from the Nernst electrode potential equations (**c** are the variables for the Nernst equation). Thus, Q′ = Q′(**c**,**n**), which is a function of the physical variables apart from the electrode potential.

For instance, let the model of the electrode be according to (3a); then differentiating (3a) with respect to Δφ yields the differential capacitance C_d_ written:

(29) $${\text{C}}_{\text{d}}=\alpha {\text{C}*}^{1/2}\text{cosh}(\beta^{\prime }\Delta \varphi /\text{RT})$$

where α are the relevant physical constants in (3a), and C* is the concentration of bulk electrolyte (moles per liter for instance), and where β′ = zF/2 (for z-z electrolytes).

Consider the equilibrium electrode equation:

(30) $$\text{AgCl}+{\text{e}}^{-}↔\text{Ag}+{\text{Cl}}^{-}$$

where Δφ = ℑ^o^ − (RT/F)ln a[Cl^−^].

Let ℑ^o^ = (RT/F)ln α′ where α′ = exp(ℑ^o^ F/RT).

Since Δφ = – (RT/F)ln (a[Cl^−^]/α′), we get

(31) $$\begin{array}{ll} {\text{C}}_{\text{d}} & =[(\alpha {\left(\text{C}*\right)}^{1/2})/2].\text{cosh}[-\beta^{\prime }/\text{F}.\;\text{ln}\;(\text{a}[{\text{Cl}}^{-}]/\alpha \prime )]\\ & =[(\alpha {\left(\text{C}*\right)}^{1/2})/2].\{{\left(\text{a}[{\text{Cl}}^{-}]/\alpha^{\prime }\right)}^{-\text{z}/2}+{\left(\text{a}[{\text{Cl}}^{-}]/\alpha \prime \right)}^{\text{z}/2}\} \end{array}$$

which is independent of the potential for the GC electrode system? Hence, if C_d_ is measured from experiment, then α′ may be determined by curve-fitting, so that the important parameter ℑ^o^ may be determined directly, if α′ does not vary appreciably with C*. Some experimental support for the above follows below. We note more elaborate expressions may be derived for extensions of the Gouy-Chapman model (e.g. that attributed to Stern) where Q the charge on the electrode is functionally written Q = f (Δφ, **n**,Q) because there is no known simple analytical function g which is a solution to f, but in principle it is always possible to write Q = g(Δφ,**n**) for the Stern and other elaborations and the above derivations (29 – 31) may be repeated using this g function.

#### 2.1.6. Lemma (6)

The concentration of an ion in dielectric 1 may be determined in terms of its concentration in another dielectric 2 which is in thermodynamic equilibrium to phase 1 by the Gibbs equilibrium criterion.

**Figure 2.**
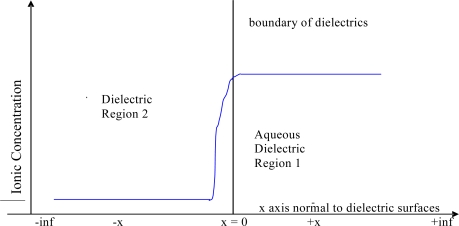
Concentration profile of ion between two dielectrics.

The two different (approximate) methods developed here are based on the assumption (Method A) that the concentration of the ion at the boundary of the two dielectric phases is that of the bulk for the phase with the higher bulk concentration, and the other method (Method B) is derived using the Born equation; m represent the bulk concentrations in molality units, ρ the densities, μ^o^ the standard potentials, γ the activities, and c the concentration in moles per unit volume (liter if numbers are quoted), and subscripts refer to the phase concerned. The assumption in Method A is inspired by the fact that the concentration of the active species is much lower in the electrode dielectric than it is in the aqueous layer, and that rapid exchange of the active ion by diffusive processes in chemical equilibrium would make the concentration near the surface close to the one for the bulk aqueous layer. The equations below apply at ‘low concentrations’ (~ 0.05 m), of the same magnitude used in the experiments reported here. Figure 2 is a representation of the system. The interface is at x = 0, with dielectrics 1 and 2 having coordinates +x and –x respectively.

### 2.2. Method A (Solution of activity coefficient equation)

The boundary conditions chosen are:

(32a) $$\underset{x\to 0}{Lim}c_{2}(-x)=\;\underset{x\to 0}{Lim}c_{1}(+x)=c_{1}(+\infty )=\rho_{1}m_{1}$$

(32b) $$\underset{x\to 0}{Lim}\gamma_{2}(-x)=\;\gamma_{2}(c_{1}(+\infty ))=\underset{x\to 0}{Lim}\gamma_{2}(m_{1}\rho_{1}/\rho_{2},-x)$$

At any plane –x and +x, the Gibbs criterion leads to the following (where the dielectrics are set at the same potential):

(33) $$\mu_{2}^{o}+RT\text{ln}\;m_{2}+RT\text{ln}\gamma_{2}=\mu_{1}^{o}+RT\text{ln}\;m_{1}+RT\text{ln}\gamma_{1}$$

At the interface, the limits (32) lead to m_1_ρ_1_ = m_2_ρ_2_ and therefore:

(34) $$\mu_{2}^{o}+\alpha -\mu_{1}^{o}=RT(\text{ln}\gamma_{1}(m_{1})-\text{ln}\gamma_{2}^{\text{int}}(\frac{m_{1}\rho_{1}}{\rho_{2}}))=\beta$$

where α = RTln(ρ_1_/ρ_2_), and β is constant for variables ±x but differs for different bulk concentrations of the ion in phase 1. Equations (33) and (34) lead to:

(35) $${\text{log}}_{10}(m_{2}\gamma_{2})={\text{log}}_{10}\left(\frac{\rho_{1}m_{1}\gamma_{2}^{\text{int}}(m_{1}\rho_{1}/\rho_{2})}{\rho_{2}}\right)$$

where $\gamma_{2}^{\text{int}}$ is the activity coefficient of the ion in the dielectric 2 at the interface; here the singularity is evident in terms of the activity coefficients, whereas the concentrations are continuous; an analogy would be as with electrostatic problems, where the voltage may be continuous but not the charge density. If we set β = 0 (implying continuity of activity coefficient), then the trivial solution m_2_ = ρ_1_m_1_/ρ_2_ ensues, implying (the absurd) conclusion that the c concentration of ions is invariant across the interface. The only approximation made here is the fixed value of the c ionic concentration at the interface; more sophisticated models might modify this concentration, as well as the value of γ_2_ at the interfacial boundary. If the permittivity of the phases follow ε_1_ > ε_2_ and c_1_ > c_2_, then the other choice of the boundary conditions is unphysical, since it would require $\underset{x\to 0}{Lim}c_{1}(+x)=\rho_{2}m_{2}$; moreover, for aqueous dielectrics, we would expect a fast interchange of ions to favor either fully or partially the given boundary conditions.

### 2.3. Method B (Approximation using the Free energy standard potential given by the Born equation)

This method is highly dependent on (a) highly accurate values of the effective radii of the ion in the dielectric and vacuum phase (to 3 decimal places), both of which need not be the same, and even until now little is known of these ionic radii under different ionic environments and concentrations and (b) the assumption that the activity coefficients at the standard states in both dielectrics 1 and 2 are approximately equal, or are small enough to be neglected, and that they are known for other ionic concentrations in the solid state dielectrics; in the relatively recent field of solid state ionics, the activity coefficients are customarily neglected by being set to unity [23] because these values are not precisely known, and they are thought not to affect significantly the calculated values of the physical properties under discussion.

The work done to convert a charged ion in vacuum (I_0_) to a state of known molality concentration I_4_ may be done in a series of stages depicted as $I_{0}\overset{\Delta G^{o}}{\to }I_{1}\overset{\Delta G_{2}}{\to }I_{2}\overset{\Delta G_{3}}{\to }I_{4}$. The energy to convert an isolated ion in vacuum to an isolated ion in the pure dielectric I_1_ is given by the Born expression:

(36) $$\Delta G^{o}=(\frac{z_{i}^{2}e^{2}N_{A}}{8\pi {ɛ}_{r}{ɛ}_{o}r_{i,vac}}-\frac{z_{i}^{2}e^{2}N_{A}}{8\pi ɛr{ɛ}_{o}r_{i,d}})$$

where *r_i,vac_* is the vacuum ionic radii and *r_i,d_* is the effective radii in the dielectric. The work done to compress the ions to unit activity at state I_2_ is q. From this state, the work done to compress the ions to any other arbitrary state I_4_ is:

(37) $$\Delta G_{3}=RT\text{ln}\;c_{i}+RT\text{ln}\gamma_{i}.$$

Thus the concentration of the ions in equilibrium between dielectric phases 1 and 2 is governed by the equation:

(38) $$\Delta G_{2}^{o}-\Delta G_{1}^{o}+q_{2}-q_{1}+RT\text{ln}\left[\frac{{\left(\rho m\gamma \right)}_{2}}{{\left(\rho m\gamma \right)}_{1}}\right]=0$$

Assuming no change in the compression energy q, the following equation obtains:

(39) $$\Delta G_{2}^{o}-\Delta G_{1}^{o}+RT\text{ln}\left[\frac{{\left(\rho m\gamma \right)}_{2}}{{\left(\rho m\gamma \right)}_{1}}\right]=0$$

(which is similar to the usual chemical equations relating the equilibrium constant to the standard free energy change, which in this case is relative to the isolated ion in vacuum).

## 3. Applications of the Above Lemmas

### 3.1. Theoretical Basis for the Esin and Markov Effects Utilizing Lemma (1)

It does not immediately follow that if (3a) is true, that the converse always obtains, where the coupled equation may lead directly to ΔΨ_d_ i.e. Q(ΔΨ_d_) ⇒ ΔΨ_d_, as is sometimes supposed [10]. For if Q(ϕ_2_)=2Asinh(|z|f ϕ_2_/2), then if | ϕ_2_ | << 1 and f is constant, the standard approximation [9] (ϕ_2_ never approaches infinity) for low ϕ_2_ fulfilling the condition (zeϕ_2_/4kT≤ 0.5) is ϕ = ϕ_2_ exp(−κx) with κ = (2nz^2^e^2^/ε.ε_o_.kT)^1/2^. If |ϕ_2_| were allowed to be very large, so that Q(ϕ_2_) ~ 2A′(n)/2 exp(|z|f ϕ_2_/2), then this value of |ϕ_2_| may not be small enough in some instances to correspond to the experimental setup where ϕ_2_ ~ (2/zf)lnQ + const - (1/zf)(ln n). The series expansion Q(ϕ_2_) = 2A{|z|f ϕ_2_ /2 +....} does not immediately yield ϕ_2_ = constant ± (1/|z|f) lnC^s^ for non-constant [10] Q as the concentration C^s^ is varied. In the cases where the e.m.f.s do not correspond and/or Q is not definitely constant, it is suggested here that the reason for the Nernst-like relation found experimentally is that the electrode is also serving as a type of self-polarizing reversible equilibrium electrode with respect to the relevant ions at bulk concentration C^s^ whilst conforming to (3) as well; and it is suggested here that these types of self-polarizing electrode systems were essentially what Frumkin et al. were studying [10]. It will be argued (with supporting experimental and numerical data) in the sections which follow that for reversible electrodes, it is a fair approximation to set x_2_ = 0, ΔΨ_d_ = Δψ_d_. For such cases, the Esin and Markov effects [10] are also explained since if we write the activity for ionic species s as a_C_^s^ = C^s^γ_±_, with bulk concentration C^s^ and (mean) activity coefficient γ_±_, leading to the identity ln a_C_^s^ = C^s^ + lnγ_±_, then at the point of zero charge, Q = 0 (for the electrode) ⇒ Δψ_d_ = 0 ⇒ c′ + E^pzc^ - (1/|z|f)ln C^s^ = 0 from the Nernst equation (neglecting activity coefficients at low concentrations) given in Lemma 4 which is logarithmic if c′ is interpreted as ‘constant’ for the electrode at that particular concentration of electrolyte with the stated physical conditions and which arises for various reasons, including a residual potential due to charges on the interface and that due to the reference electrode, and of course, the presence of a mean activity coefficient which may be absorbed by the Nernst equation by the c′ term at higher concentrations. And in general if ΔΨ_d_ = ΔΨ_d_ (Δψ_d_), a first-order Taylor expansion at the point of zero charge (ΔΨ_d_ = 0) implies a + bΔΨ_d_ = 0 (a,b are constants) or c” + 1/b(E’^pzc^ - (1/|z|f)lnC^S^) = 0 and logarithmic behavior would still be observed under the assumption that these systems are essentially functioning as reversible (self-polarizing) electrodes, as discussed in Section II, Lemma (1); E^pzc^,E’^pzc^ and c” are all constants.

It appears that both the usual sinh expansion and/or Lemma 1 may be utilized to partially explain the many experimental phenomena connected with potentials of zero charge and the electrode interface. However, there is a way to distinguish the two rationalizations by carrying out measurements in a regime for which the sinh expansion is not valid.

**Figure 3.**
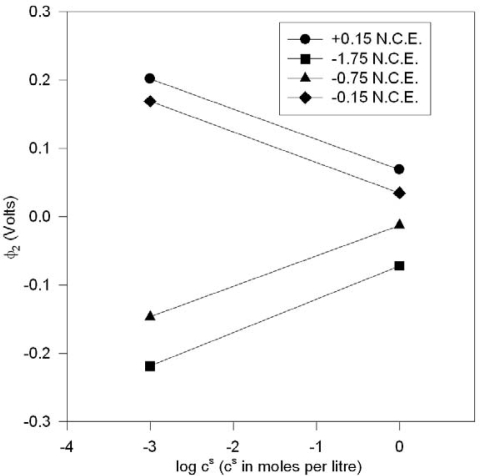
Variation of ϕ_2_ with bulk ionic concentration.

If logarithmic behavior is still observed in these non-valid regimes, then it appears probable that the standard approximate relationship mentioned above may be open to question. The available data [10] as depicted by the plots in Figure 3 seems to suggest logarithmic behavior outside the region of validity of the sinh expansion. Figure 3 are plots of the ϕ_2_ (potential of the plane of closest approach of the solution electrolyte to the mercury electrode surface relative to the bulk sodium fluoride concentration c^s^) against the logarithm of the bulk concentration of NaF for various potentials of the mercury electrode relative to the Normal calomel reference electrode (N.C.E.).

In the conventional analysis, the ϕ_2_ value as predicted by the conventional theories obtain when [10] |ϕ_2_ | ≥ 0.1 V, but it is evident that linearity of the plots in Figure 3, derived from experimental data [10] also obtain outside of the stated regime, and therefore indicates a possible need for another explanation, such as what has been attempted here. In the notation of Figure 3, ϕ_2_ ≈ Δψ_d_ when x_2_ = 0.

### 3.2. Estimation of Solution Standard Potential from Lemma (4) for the Ag|AgCl Electrode

The value of the tenth-normal Ag|AgCl electrode potential is approximately 0.5125 V at this maximum [24] which implies at this concentration that the average ℑ^o^ ~ 0.512 V for aq. NaCl. Relative to the calomel electrode, the value is 0.557 V for 0.1 to 1.0 N solutions [25], implying that the electrochemical standard potential for the hydrogen electrode accounts accurately for this difference (~ 0.045 V) between the Ag|AgCl and Hg|HgCl (or Hg_2_Cl_2_) electrodes (of Standard Potentials 0.2223 V and 0.2680 V respectively with difference 0.0457 V). Hence, for aq. KCl, we may infer with confidence the approximate ℑ^o^ value from the e.c.m. value relative to the calomel electrode by subtraction of ~ 0.045 V (the difference of these standard potentials) since no comparable data are readily available for the e.c.m. potential for KCl relative to the Ag|AgCl electrode. From the data [25], there is a clear logarithmic behavior of the e.c.m. potential for low concentrations (0.001 to 0.1 N KCl solution). Relative to the Ag|AgCl electrode, we derive ℑ^o^ = 0.4489 V for the lower limit for ℑ^o^ since at higher concentrations a charge transfer from the external cell modifies ℑ^o^. Higher values of ℑ^o^ would exaggerate the effects of the computed e.m.f. for cell (1a), calculated according to the prescription given in Section V below. The more accurate value for ℑ^o^ must be derived taking into account the spread of values over all concentration ranges of the electrolyte, from the constant value regime of ~ 0.557 V for 0.1 to 1.0 N solutions to the logarithmic regime at very low concentrations as determined for instance by the classic results of Grahame and others (Table 1 of reference [25]). At higher concentrations an extraneous e.m.f. is required to force electrons into the mercury electrode as well as withdraw electrons from the Ag|AgCl electrode leading to a more positive value ℑ^str^ for ℑ^o^, where ℑ^str^ denotes the ‘stressed’ value, so that the measured value of the e.m.f. at the e.c.m. of Hg, E_m_ may be written:

(40a) $${\text{E}}_{\text{m}}={ℑ}^{\text{str}}-(\text{RT}/\text{F})\text{ln}\;\text{a}[{\text{Cl}}^{-}]$$

where a[Cl^−^] is the activity of Cl^−^ ions, and we also define:

(40b) $$\Delta \varphi ={ℑ}^{\text{O}}-\;(\text{RT}/\text{F})\text{ln}\;\text{a}[{\text{Cl}}^{-}]$$

where when E_m_ → Δϕ, ℑ^str^ → ℑ^o^. The general Taylor expansion for ℑ^str^ with these conditions are:

(40c) $$\Delta ℑ={ℑ}^{\text{str}}-{ℑ}^{\text{O}}={\left({\text{E}}_{\text{m}}-\Delta \varphi \right)}^{{\text{i}}_{{\text{a}}_{\text{i}}}}$$

where an obvious solution of (40c) is:

(40d) $${\text{E}}_{\text{m}}={ℑ}^{\text{str}}-(\text{RT}/\text{F})\;\text{ln}\;\text{a}[{\text{Cl}}^{-}]$$

where a_i_ = 0 for i ≠ 1, and a_1_ = 1.

At the constant E_m_ regime, we note that a compensating (−RT/F)ln(a) factor to the strained value ℑ^str^ leads to a constant, so that a solution to this regime is:

(40e) $${ℑ}^{\text{str}}={ℑ}^{\text{O}}-({\text{c}}^{\prime }-(\text{RT}/\text{F})\text{ln}\;\text{a}[{\text{Cl}}^{-}])$$

with c′ a constant. If c′ = 0, then E_m_ = ℑ^o^. On the other hand, (as explained below) at the logarithmic phase, E_m_ → Δφ, ℑ^str^ → ℑ^o^, so that:

(40f) $${\text{E}}_{\text{m}}={ℑ}^{\text{O}}-(\text{RT}/\text{F})\;\text{ln}\;\text{a}[{\text{Cl}}^{-}]$$

If the change of regime is reasonably abrupt, then at a[Cl^−^] = a_crit_, the critical activity, both (40e) and (40f) are jointly satisfied, and thus:

(40g) $${ℑ}^{\text{O}}-(\text{RT}/\text{F})\;\text{ln}\;({\text{a}}_{\text{crit}})={ℑ}^{\text{O}}-({\text{c}}^{\prime }\;-\;(\text{RT}/\text{F})\;\text{ln}\;({\text{a}}_{\text{crit}}\text{)})-\;(\text{RT}/\text{F})\;\text{ln}\;({\text{a}}_{\text{crit}})$$

⇒ c′ = (RT/F) ln(a_crit_) and:

(40h) $${\text{E}}_{\text{m},\text{crit}}+(\text{RT}/\text{F})\text{ln}\;({\text{a}}_{\text{crit}})={ℑ}^{\text{O}}$$

From the data [25,26] the critical value of the concentration is 0.1 N for KCl, leading to the value of ℑ^o^ = 0.4489 V, which is incidentally somewhat close to the ‘rational’ potential of Grahame [27] set at 0.480 V for the Hg (and other electrodes). If this rational potential of Grahame is used for ℑ^o^, the computed activity coefficient results given in the Tables of the Appendix would show even more striking departure from the experimental values. We have assumed activity coefficient from Pitzer’s single ion activity coefficient of 0.7672 for this concentration (0.1 N) for the chloride ion (details below). A compendium of Grahame’s results [10] give the e.c.m. voltage relative to the N.C.E. as 0.505 V at 25 °C for 0.1 N KCl, or 0.4600 V relative to the Ag|AgCl electrode. From (40a), we derive ℑ|_.1N_ = 0.394031 V which will be the value chosen to solve the coupled electrode differential equations.

### 3.3. Postulated Nature of the Hg Electrode at the e.c.m Based on Lemma (4)

Some writers have viewed the Hg electrode as a reversible cell [28] and constructed theories whereby Hg_a_ and Hg_b_, the activities corresponding to the electrodes of the half-cells might cancel even if they are at different potentials and this construct demands amongst other things that such species as (KCl)_a_ [28] is not activity dependent. Overwhelmingly, however, the surface of this electrode is treated using the Gibbs adsorption isotherm [10,29]. Another view presented here is that over an arbitrary distance δl from the surface, we may envisage two types of processes occurring at extremes of concentrations:

(i) At sufficiently high concentrations of bulk solution electrolyte, the concentration of ions adsorbed within distance δl (where δl extends to a specified distance where negligible amounts of ions are present and which is expected from the Faraday demonstration of charges residing at the surface of the conductor) is approximately linear to first order in the bulk solution strength where the usual Gibbsian equilibrium conditions prevail written:

(41) $$\mu^{\text{O}}(\text{Hg},{\text{z}}_{\pm })+{\text{z}}_{\pm }\text{F}\varphi (\text{Hg})+(\text{RT}/\text{F})\text{ln}\;({\text{km}}_{\pm })=\mu^{\text{O}}(\text{s},{\text{z}}_{\pm })+(\text{RT}/\text{F})\text{ln}\;({\text{m}}_{\pm })+{\text{z}}_{\pm }\text{F}\varphi_{\text{S}}$$

where k is the proportionality factor, z the signed charge of the species and the ± refers to the positive or negative charge of the adsorbed species. The linearity assumption follows from continuity and the steep concentration gradients from bulk to the surface of the Hg electrode, and reversibility here refers to the thermodynamical condition that all thermodynamical equilibrium states are reversible states so that state functions may be traced out. At these concentrations, the Hg electrode can function as a reversible electrode to anions, cations or both, depending on the experimental and theoretical situation. We give an example of anion exchange. At this regime, the impressed external e.m.f. changes or moderates the “standard” potential ℑ but the charges transferred by the seat of e.m.f. into the cell is insufficient to change it into an inert metal electrode. We can envisage, for example for all E_m_ values up to and including the e.c.m. the following electrode reaction:

(42) $${{\text{Na}}^{+}}_{{\text{x}}^{.}}\text{Hg}({\text{Cl}}^{-})+\text{e}↔{\text{Cl}}^{-}+\text{Hg}({\text{e}}^{-}).{{\text{Na}}^{+}}_{\text{x}}$$

Applying the Gibbs criterion yields the electrode solution potential Δφ_Hg-S_ as:

(43) $$\begin{array}{l} \Delta \varphi_{\text{Hg}-\text{S}}=\{\mu (\text{Hg}(\text{e}))+\mu (\text{Na}+\text{x}.\text{Hg}(\text{Cl}-))-\mu (\text{Hg}(\text{e}).\text{Na}+\text{x})-\mu^{\text{O}}({\text{Cl}}^{-})-\text{RTln}\;\text{a}[{\text{Cl}}^{-}]\}/\text{F}\\ \;\;\;\;\;\;\;\;\;\;\;={{ℑ}^{\text{str}}}_{\text{Hg}}({\text{E}}_{\text{m}})-(\text{RT}/\text{F})\text{lna}[{\text{Cl}}^{-}] \end{array}$$

Hence the total cell potential difference for the Hg and Ag|AgCl electrodes becomes:

(44) $${\text{E}}_{\text{m}}={ℑ}^{\text{O}}-{{ℑ}^{\text{str}}}_{\text{Hg}}$$

However, at the e.c.m, the elementary Lippman theory in conjunction with the Gouy-Chapman description demands Δφ_Hg-S_ = 0, so that ℑ^str^_Hg_ (E_m_) = RT/Fln a[Cl^−^] and thus (44) becomes:

(45a) $${\text{E}}_{\text{m}}={{ℑ}^{\text{str}}}_{\text{Ag}|{\text{AgCl}}^{-}}(\text{RT}/\text{F})\text{ln}\;\text{a}[{\text{Cl}}^{-}]$$

which is precisely (40f) at the e.c.m.. Cationic (or both) exchange does not alter the basic features of the argument. At the e.c.m., we can infer that Na^+^, Cl^−^, and e^−^ charges exists within the electrode to the extent that the net charge is zero.

(ii) At very low concentrations, the reversible anion or cation mechanism breaks down and the electrode may be viewed as “inert” but as having the same potential as the solution within the limits of the Gouy-Chapman theory of electrodes since residual charges and the capacitance becomes arbitrarily small as the concentration drops. Hence:

(45b) $${\text{E}}_{\text{m}}\approx {{ℑ}^{\text{O}}}_{\text{Ag}|{\text{AgCl}}^{-}}(\text{RT}/\text{F})\text{ln}\;\text{a}[{\text{Cl}}^{-}]$$

and logarithmic behavior is predicted at this regime, as observed also in experiment.

### 3.4. Application of Lemma (5) to Grahame’s Experimental Observations of the Constancy of the E.C.M. E.M.F. at Higher Electrolyte Concentration

**Figure 4.**
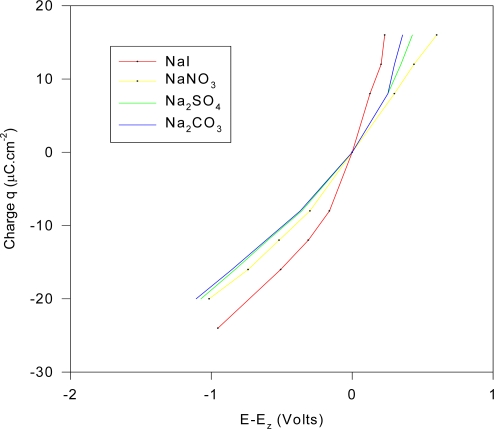
Charge on Hg in contact with 1M electrolytes at 25 °C.

(1) Another gross verification of the present capacitance theorem may be observed from the experimental data provided by Grahame [27,30,31], some of which are sketched in Figure 4, which includes positive values of the Hg interfacial potential. Grahame also provides similar data for an additional 9 sodium salts [30,31] at negative values of the double layer potential, where the lines all have the same slope of ~ 17 μFcm^−2^. We note in Figure 4 that for strong electrolytes (at 1 M solutions), the plot of charge against the potential relative to that at zero charge are quite linear for especially the negative potential and of the same gradient of ~ 17 μFcm^−2^. Moreover, they all coincide at the point of zero charge. For a single-species ionic equilibria:

(45c) $${\text{M}}^{\text{z}+}+{\text{ze}}^{-}\to \text{M}$$

we can expect for the same species M^z+^ approximately the same value for the C_d_ capacitance from equation (31) for the same bulk cationic concentration if (45c) is the predominant electrode reaction. If the Hg interfacial potential (E-E_z_) were polarized negative, it would encourage the cationic (45c) exchange reaction since species M would adsorb to the Hg interface according to such mechanisms as Hg + xNa^+^(aq) → Hg.x(Na^+^)|_electrode_ and that described by (42). The determination of standard potentials for highly reactive metallic species in aqueous solution has been accomplished by Hg amalgam electrodes, which the above system might approximate at relatively high negative potentials. These conclusions accord with the data, such as depicted in Figure 4 for constant 1 M electrolytes with counterions I^−^, NO_3_^−^, SO_4_^2−^ and CO_3_^2−^ with the approximate capacitance of 17 μFcm^−2^ with the interfacial voltage (relative to the N.C.E) of ~ 0.4 to −1.2 V. This capacitance does not obtain for the positively charged surface (~ 0 to + 0.6 V) and it is conjectured that the reason for this is because (45c) ceases to be the dominant electrode reaction at such positive potentials, because of the expulsion of the cations from the Hg interface.

If the mercury is envisaged as an electrode reversible to ions as outlined in (1) above and explained below, then, we might expect such a close fitting linearity (assuming similar values of activities) since C_d_ ~ constant for a fixed concentration of aqueous electrolyte, leading to the same gradients for the different curves. The parallel nature of the lines are due to changes in the physical conditions due to the different counter ions. For NaI, the curve is crudely of the form sinh(κφ) (constant κ, with potential φ), implying a net ideally polarizable inert electrode with non-reversibility of ions (no charge transfer).

(2) Concerning the constancy of the e.c.m. voltage, Grahame remarks [32]that “one sees at once a remarkable constancy which could not be anticipated”, and relative to his theories, “reflects an astonishingly constant effective radius at the interface.” There are actually two constancies observed, a′) for any one electrolyte, at moderate concentrations (0.1 to 1.0 N), the e.c.m. voltage is invariant and b′) for all the cited electrolytes, the e.c.m. voltage is remarkably constant, with median value of 0.5588 V. For situation a′), we suppose that the measured e.m.f. E when E_m_ = 0 (i.e. at a value not corresponding to the e.c.m.) for the case of net Cl^−^ reversibility that:

(46) $$\text{E}={ℑ}^{\text{O}\;-}{{ℑ}^{\text{O}}}_{\text{Hg}}(\text{M})$$

where ℑ^o^_Hg_(M) is a standard potential at molality M. Now, if the impressed voltage E_m_ conserves charge within its own circuitry, then the charge Q_ref_ extracted from the reference electrode (e.g.Ag|AgCl) equals that which is injected into the Hg electrode Q_Hg_, i.e. Q_ref_ = Q_Hg_. The change in potential Δφ′_ref_ for the reference electrode is Cd.Δφ′_ref_ = Q_ref_ since electrons are withdrawn. If we postulate that the impressed voltage on the reference electrode makes it more positive with respect to the solution, then from the reaction equilibria:

(47a) $$\text{AgCl}\;+\;\text{e}\;↔\;\text{Ag}\;+\;{\text{Cl}}^{-}$$

we anticipate the absorption of Cl^−^ from the solution. Likewise, we observe that there is a net negative charge on the Hg electrode (relative to the reference electrode) and hence from (43) and (44), there will be an expulsion of Cl^−^ ions to the solution, leading to a neutral electrode (Δφ_Hg-S_ = 0) at the e.c.m..Hence, relative to the solution, the effect is the raising of the potential of the electrode. Another way of viewing this is to suppose the net absorption of Na^+^ ions (so that the solution remains uncharged) by the influence of the negative potential, which is written as:

(47b) $$\text{Hg}\;+\;{\text{xNa}}^{+}(\text{aq})\;\to \text{Hg}.\text{x}({\text{Na}}^{+})\;(\text{electrode})$$

In either case, the increase of potential Δφ′_Hg-S_ relative to the solution due to the ionic interchange is such that Cd. Δφ′_Hg-S_ = Q_ref_, so that Δφ′_Hg-S_ = Δφ′_ref_.

Hence at all moderate concentrations:

(47c) $${\text{E}}_{\text{m}}\approx \text{constant}\approx \text{E}.$$

**Figure 5.**
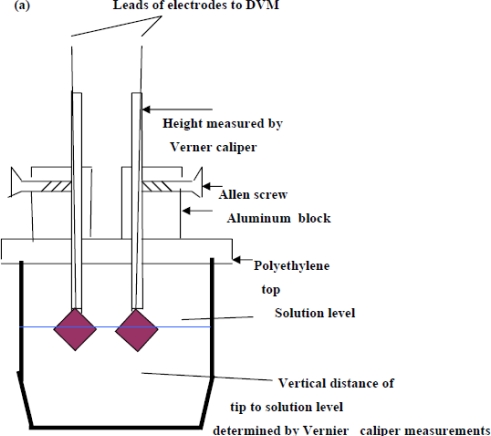

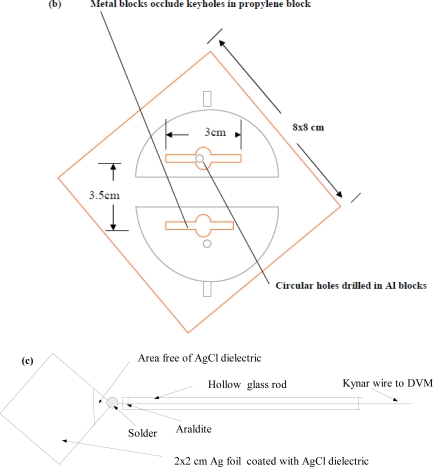
(a) General setup of cell. (b) Plan view of square polyethylene top for electrode; (c) Electrode used in the experiments.

The similarity of value of potential in b′) for different electrolytes may be explained by postulating that the k values in (41 – 42) are approximately the same for each of the different types of anions (k_a_) and cations (k_c_), where k_a_ is not necessarily equal to k_c_ for strong electrolytes, and they might be treated as point charges independent of size within the surface of the mercury electrode. The above is clearly only an outline and other alternative net mechanisms may be adduced. Figures 5(a – c) display a schematic of the experimental system.

## 4. Experimental Details of Low Resolution Cell E.M.F. Measurement

The setup of the system used for a low resolution experiment to determine e.m.f. contributions due to size effects is given in Figures 5(a – c). We reiterate that all the other experiments were done by established pioneers in this field.

Silver foil (Aldrich Chemicals, 99.98% purity) of square dimension 2 × 2 cm and thickness 0.025 mm and 1 mm were used for electrodes s2 and s1 respectively found in (1a). It was found that massive or heavy electrode holders or wiring [meaning ~2 gm (grams) excess weight for brass connectors] could alter the measured e.m.f. by ~ 0.5 mV if one of the electrodes were connected to such an arrangement. To avoid possible capacitive effects due to unequal metal mass contact, and also sheer mass of the connectors, the electrodes were directly spot soldered at one of the corners, (Figure 5(c)) and the bare Ag and solder were painted with a non-conducting polymer to avoid corrosion effects, where very fine Kynar (U.S.A.) AWG #30 wrapping wire, 23 cm in length served as leads.

The Ag foil electrodes were all electrolyzed under identical conditions, and the temperature was maintained at 25 ± 0.02 °C. This electrolysis to create the AgCl layer was carried out at 7.8830 mA for 1,200 seconds in HCl solution (1.5 M) using two platinum coated titanium gauzes as counterelectrodes for all electrodes s2 (under identical conditions) and the current corresponds to a total thickness of 2.5 μm (microns) of Ag which was converted to AgCl. The thickness mentioned refer to the Ag substrate and not to the AgCl deposit. The density of Ag was taken to be 10.501 gm cm^–3^ at 25 °C. The slight reduction in the average thickness of the Ag substrate was accounted for in the computations. The electrodes were dry weighed before and after electrolysis by a Mettler MT5 microbalance to determine the efficiency of conversion at ~ 92 ± 5%. The electrolysis utilized a commercially available potentiostat [EG & G Princeton Applied Research 363 Potentiostat (U.S.A)], and all e.m.f. measurements were made using a digital voltmeter [Keithley 197A Autoranging μV DMM (U.S.A.)]. Over 60 readings of cell e.m.f. E_c_ = ϕ_10_ – ϕ_20_ (where ϕ_j0_ represents the potential of the Ag substrate j) were taken each lasting at least 1/2 hour; further details of the measurements follow below. One result (without exception) was that E_c_ was negative to the order exceeding 1 mV for all the 60 measurements used in the e.m.f. determination under controlled conditions. The KCl solution was prepared from Analar grade KCl and doubly distilled water by weighing (since molality units were used). For the ordered set of solution concentrations m (molality units) {0.01, 0.03, 0.05, 0.07, 0.1}, the corresponding ordered set of averaged - E_c_ values (units of mV [millivolt], with a maximum experimental fluctuation per reading of ± 0.2 mV) are {1.46, 1.51, 1.57, 1.56, 1.77}. Altogether, 24 separate s2 electrodes were fabricated and electrolyzed, and studied and used for the qualitative and quantitative studies.

**Figure 6.**
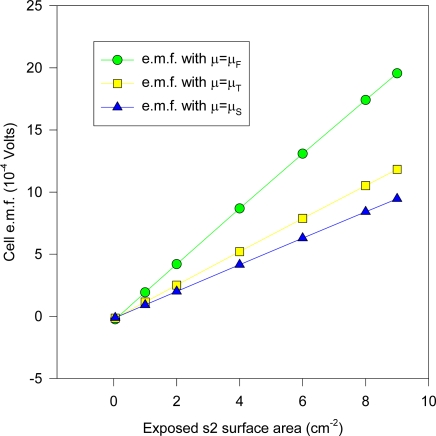
Variation of cell e.m.f. with area of electrode s2 exposed to 0.05 m aq. KCl.

Many of these electrodes were cleaned and re-electrolyzed to confirm the observations concerning the AgCl layer and the e.m.f. measurements. The measurements for different KCl concentrations were done slowly over a 10 month period (December 1997-September 1998), where two separate s1 electrodes were fabricated and used. The estimated cell e.m.f. observational errors is based on this relatively long time period of general experimental. It is deduced here that the inaccuracies are mainly attributable to the method of fabricating the electrodes and the inhomogeneity of the electrode materials, as discussed in the text. The cell e.m.f. results presented for any one KCl concentration is the average of measurements carried out at least twice at different times from different stock solutions. The input resistance of the Keithley instrument was > 1 GΩ for the range of e.m.f. measured, and so internal i-r drops and other associated effects causing extraneous e.m.f.s may be safely discounted. Further, the electrode e.m.f. was reset to zero volts by contact of the upper unelectrolyzed part of the electrode before immersing s2 into the solution, which would eliminate polarization e.m.f.s due to the circuitry other than the electrode system. That i-r drops are negligible for these measurements may also be inferred form the fact that no discernable change in e.m.f. (of 1 μV order) could be detected when distance between electrodes s1 and s2 were varied (to ~ 4 cm). Also, the observed e.m.f. polarity was the same for all of the measurements of the s1 – s2 couple, implying an intrinsic effect due to electrode size effects only. To confirm that the electrode e.m.f. differences were due to the Ag substrate size differences and not due to electrochemical differences caused by different coating thicknesses of the AgCl dielectric, five of the s2 electrodes were electrolyzed at half the current intensity for the same time (corresponding to total thickness of 1.25 μm of Ag converted to AgCl). It was experimentally found that the effects due to coating thickness was effectively negligible where the measured e.m.f. of cell (1a) is concerned, which accords with the common and fundamental electrochemical assumption (such as used in (2a)) where the solid dielectric or salt activity (in this case AgCl) is assigned value unity, with constant chemical potential for all electrodes regardless of amount. The surface area of s1 exposed to the solution was maintained constant, corresponding to a penetration depth (into the KCl solution measured from the tip of the electrode to the Ag-AgCl interface) of 1.920 cm measured by Vernier caliper, with an exposed solution area of approximately 6.90 cm^2^, but s2 varied by ~ 6 ± 0.5 cm^2^ because the electrolysis involved vigorous stirring, so that the Ag-AgCl line boundary could not be exactly fixed; so all readings were normalized to 8 cm^2^ surface area exposure for s2 by linear extrapolation with s1 fixed with the stated extent of surface area. That this is a reasonable extrapolation may be inferred from Figure 6 for a theoretical calculation of the change of cell potential with the area of electrode s2 immersed in KCl (aq) for fixed s1 penetration depth (1.920 cm). In Figure 6, the chemical potentials are as described previously where μ_S_ = ε_o_ + d[nε_xc_(n)]/dn (Equation 16), μ_T_(e) = μ_F_(e)+ ε_xc_. (equation. 17) and μ_F_(e) (equation. 5). The method of calculating the charge transfer and potential is given immediately below in greater detail in Sec. 5.1 entitled computational method for cell e.m f. after first describing the experimental setup. The theoretical implications are discussed after Sec. 5.1.

This calculation was verified qualitatively by experiment, but because of the porosity of the AgCl dielectric, coupled with the effects of a solution film still present in areas not immersed in the solution, direct verification is difficult, but an e.m.f. change of ~ 0.5mV could be detected as a result of change in the degree of penetration of the s2 electrode into the KCl solution. The surface area are in units of square centimeter of electrode exposed to the reactive solution, and the potential that develops as a result of the charge transfer from the AgCl dielectric in contact with the solution.

Interestingly enough, a direct confirmation of this result is to be found in the award-winning work of K.L. Cheng [33]. Here, Cheng presents results of varying accuracy of ion selective electrodes where the cell e.m.f. varies quite linearly with fraction of immersion depth (which may be translated as being proportional to surface area exposed for tubular electrodes), where in particular, the glass electrode receives special attention [33] (e.g. his Figure 5). This type of electrode [34] may be represented by the diagram: Reference electrode, X, H^+^ | Glass; where X represents the ion to which the reference electrode is reversible; the reference electrode is here defined to be the ‘inner electrode’ for this system. It must be pointed out that whilst the experimental results of Cheng concerning linear change of e.m.f. with exposed surface area of the electrode agree in terms of predicted effect for the specialized case of our Ag/AgCl electrodes, there are some theoretical differences. Cheng in particular seems to believe that the “Nernst equation has been misused” in nonfaradaic potentiometry, where “nonfaradaic processes deal with neither redox reactions nor current production”. Where only the glass membrane itself is concerned, Cheng’s analysis seems plausible by treating it as a linear capacitor (i.e. linear relation of charge and voltage), where the extent of adsorption of ions on the glass dielectric surface determines the net charge and voltage. Clearly, the ‘Nernst-like’ behavior, (which is theoretically derived from the Gibbs equilibrium criterion) of the electrodes in all probability originates form the coupling between the induced charge on the membrane, and the ion X which is reversible to the inner electrode. Cheng seems not to focus his attention nor his experiments on the inner reference electrode mechanism and the ions reversible to it (which he seems to treat as a black box potential sensor with no interfering role), but instead concentrates on the membrane potentials which are assumed to derive from the “activities” of the inner and outer layer of the membrane surfaces {his Equations (9–10) of reference [27]}. In the theoretical computations depicted in Figure 6, we first note that a second-order effect is being computed [the normal electrochemical assumption implies zero e.m.f. for the setup in (1a)], which differs from the arrangement used in Cheng’s studies where the reactive electrolytes and/or reference electrodes are not of the same type or kind. Second, the high degree of linearity observed is due to the net effect of completely non-linear coupled phenomena involving a) the validity of the Nernst equation, or equivalently the Gibbs equilibrium criterion where electrode-solution reactions are concerned, b) the use of the non-linear electronic chemical potential, and c) the utilization of the GC theory or the Stern modification for the interfacial region. The method for the solution of this problem is given in Section V. The net effect seems to be somewhat like that found in a linear capacitor, with the metal substrate playing the major role as an electron sump, leading to the high degree of linearity. Similarly, it is conjectured here that Cheng’s highly linear experimental results for the glass electrode is due to the combined linear capacitance effect (which he mentions almost exclusively) of the glass membrane and the Nernst-like behavior of the inner reference electrode as it exchanges ions with the inner glass membrane. For departures from Nernst-like behavior, apart from reasons already mentioned by Cheng, one would expect (especially at very low pH) corrosive side-reactions that would destroy the molecular glass interfacial structure and upset its capacitive linearity. Notwithstanding, Cheng’s very interesting experimental results lend further support to the linearity assumption used here (based on theoretical calculations) in normalizing the electrodes in terms of fixed surface area.

For the bulky s1 electrode, calculations show that the cell e.m.f. varies by only ~ 0.02 mV for a 4 cm^2^ surface area change for a totally immersed s2 electrode, as expected. Hence it is sufficient to normalize just electrode s2. The normalization scale factor magnifies the raw experimental results by only an approximately further 0.3 mV. To conserve costs and to remove systematic errors, only one reference electrode s1 (0.1 cm thickness) was used for all the measurements involving s2. However, to check for consistency, another s1 electrode was made and immersed to the same extent into the solution, and after ½ hr (hour) equilibration, a maximum e.m.f. difference of 0.127 mV was observed between the two electrodes at 0.1 m KCl, in contrast to the value ~ 1.2 mV detected without fail when s2 is substituted. All the electrodes were stored in a solution comprising 0.05 M KCl and 0.05 M HCl. All e.m.f. cell measurements were conducted at the solution temperature of 25 ± 0.02 °C using a precision temperature controlled cooling unit with an inbuilt water circulation pump (Polyscience^®^ Temperature Control Unit (U.S.A.)), where the cell was placed in a lagged glass cooling jacket. The depth of penetration of the electrodes was measured by means of a vernier caliper (±0.01 mm) by observing the change of vertical distance of the upper flat surface of the Aluminum holder to a point marked on the glass rod of the electrode when the pointed electrode corner tip just touches the solution to the position when the electrode is at rest with the solution level coinciding with the boundary line between the AgCl dielectric and that of the pure Ag surface (Figure 5(c)). The measurements were for ambient conditions without N_2_ degassing, since N_2_ degassing will increase the pH of the solution (normally at ~ 4.42 for the range 0.01 to 0.1 M KCl) to well over 7 because of the expulsion of CO_2_ gas which forms an acidic solution in water; from the Pourbaix diagram [35] Ag_2_O formation (line (6) of reference (7)) is encouraged when the pH exceeds 6.5. Apart from reasons connected with Grahame’s extensive experimental data and acute observations and the tendency of bare Ag surfaces to form oxides in higher solution pH’s, the Ag/AgCl electrode was chosen for study because the internal Ag/AgCl interface would be less prone to form the silver oxides by the occlusion of the Ag surfaces by the AgCl dielectric, and possibly also because of its storage in an acidic medium which will discourage oxide formation provided the relevant ionic interchanges occur in the AgCl dielectric. Nevertheless, a slight drift of ~ 0.3 mV was noticed in a time period of several ½ hours, which may be attributed to oxide and other side-reactions. Thus, after a measurement, the electrode was returned to the acidic storage solution and left there for at least 24 hrs before the electrode is reused. The criteria used here to record a result is that the electrodes must be in equilibrium for at least ½ hour, and that the e.m.f. reading remains unchanged to 1 μV for at least ½ minute; and 4 – 7 different electrodes were used for each of the solution strengths indicated, and they all gave fairly good agreement according to the errors quoted. It is difficult to specify the contribution of the leads to the lowering of the cell e.m.f. but based on weight, we expect it to be less that 0.2 mV. Hence the readings given reflect order of magnitude effects, which provides a guide to the creation of theories, but a fully quantitative theory would require more stringent specifications of the solution pH and the partial pressures of the gasses in equilibrium with the solution, as well as the capacitance contribution of the leads to the electrodes, which will be the object for further studies.

The cell was constructed from a 250 mL glass beaker (of diameter 7.0 cm) cut to approximate height 7.8 cm, where the KCl test solution reached a height of ~ 3.5 cm. The length of the hollow soft glass (0.56 cm diameter) used in the electrodes was 14.5 cm. The electrodes s1 and s2 were placed at a fixed distance of 3.5 cm, and where aligned so that their major surfaces were parallel because it was observed through trial and error that for at least this configuration, there was no detectable e.m.f. change as the distance of the electrodes varied (due to the solution ir drop caused by the external measuring circuitry). Two large aluminum holders of approximate height 3.4 cm and diameter 3.8 cm with a hole in the center to accommodate the electrodes, which were held by the pressure of Allen screws tapped at the sides of the blocks, both of which were planed at one vertical side (see Figure 5(b)) to allow for a closer interelectrode distance. The polypropylene top of thickness 7.5 mm for the cell was machined with a groove to fit the glass cell and two keyholes were constructed so that the electrodes could be inserted into the cell and fastened in place by the holders efficiently without undue disturbance. The keyholes were completely occluded by the metal blocks.

The experimental outcome for the above series of experiments, derived over a 10-month period after repeated trials with many samples is reported in the figures of Section 5 together with the theoretical predictions. Some comments concerning the accuracy of these measurements are in order, together with the reasons why these measurements were made over a range of KCl solution concentrations. Firstly, this experiment was aimed at detecting second-order effects which are assumed to be non-existent in normal electrochemical practice, where the measured cell e.m.f.s are typically 50 – 100 times greater in magnitude to that presented here, with error margins of the same magnitude as the averaged results given here, because in normal practice, size is not a controlled variable, and the variation in size for the different electrodes is approximately of the same magnitude as that between electrode s1 and s2, leading to uncertainty in the region of some fraction of mV, to approximately 1.0 mV; here we have controlled the geometrical structure of the electrodes, and the polarity of the e.m.f. was the same and the magnitude of the e.m.f. was approximately the same for all 60 readings that were made, implying a size effect. If no size effect were present, then the polarity of the measured e.m.f. would vary randomly, and this was not observed at all. The measurements over different KCl solution concentrations were done to verify the curious ‘non-Nernstian’ effect predicted by the theory, where the minute change of the e.m.f. for the cell due to change in the KCl solution concentration (from 0.01 m to 0.10 m) was in the order of 0.13 – 0.06 mV (depending on the type of chemical potential used). A typically Nernstian result would be in the order of 2.303 RT/F Volts (~59.2 mV at 25 °C), larger by a factor of ~700. This theoretical prediction is borne out in the experiments, where there was no dramatic change in the e.m.f. over the stated range of KCl concentrations. It was never the intention of the experiment to attempt to map out the actual change of the e.m.f. with concentration; the errors in observation would preclude that, although there is a hint, based on average values, of a very slight general increase which may not be considered statistically significant at all. The main intention was to detect a size dependent, fairly stable and constant e.m.f. presence in a variety of conditions, such as varying electrolyte concentration, so as to rule out other factors which might cause these changes. Another advantage of measuring over a concentration range is to give an indication of the most appropriate electronic chemical potential of the substrate electrodes, since the predicted e.m.f. differences based on the different chemical potential models are greater than the errors of measurement; i.e. the band (or spread) of experimental e.m.f. values over the concentration range can give an indication of the most appropriate theoretical model of the chemical potential. The results obtained here seem to especially support the free-electron gas chemical potential, which is corroborated by the low-temperature experimental specific heat capacity results for Ag.

Lastly, can the accuracy of the experiments be further improved by suitable precautions? It may be improved, but the precautions taken would probably involve methods and facilities that go beyond the techniques routinely employed in college Electrochemical and Analytical laboratories and the experimental capabilities and facilities of our laboratories. For instance, it is known [36] that the nature of the silver halide films that are deposited by electrolysis is current dependent; at current densities less than 10 mA cm^−2^ (milliAmperes per square centimeter) in 0.1N halide solutions the deposits observed under an electron microscope are non-porous, whereas at other current densities, other different growth mechanisms are observed, with the deposited halide films having different physical properties (such as the specific conductance) and hence the average chemical potential of these substances would be expected to vary for each of the different halide structures. Under normal aqueous electrolysis, the field gradients would vary with factors such as the position of the counter-electrodes and the turbulent motion of the vigorously stirred solution; with a magnetic stirrer, one can achieve an “averaged” halide film structure, but there will be fluctuations about the mean for the different electrodes that will cause slightly different e.m.f. readings. (It is also very difficult to control the height of the AgCl/Ag interface because of the turbulent electrolysis, and clearly this is one source of the varying e.m.f. because of the different surface areas exposed.) In principle, one way of overcoming the problem of irregular film physical properties is to utilize some form of high temperature vapor deposition of the AgCl layer on the same form of Ag surfaces. The commercially procured Ag foils were presumably milled, implying again an average surface structure which could contribute to the e.m.f. fluctuations; in principle this problem could be overcome by using silver sheets with fixed crystal orientation, which are not readily available, if at all. There is also the problem of spot soldering or welding. The amount used would vary unless equally weighted amounts were used for all the electrodes. Then again, depending on the Ag crystal orientation of the metallic grains, one could expect small differences in the Volta contact potentials, leading to very small possible e.m.f. fluctuations. Although Analar reagents were used, it has been demonstrated that in normal ‘first order’ electrochemistry, trace amounts [36] (of % concentrations lower than found in Analytical reagents) can affect the e.m.f. of cells minutely (0.05 – 0.2 mV), and they could be introduced from several sources, such as the electrodes themselves. The effect of these trace ions would be expected to be less dramatic, but nevertheless there would be some interference of unknown magnitude less than 0.05 – 0.2mV. The cell e.m.f. could possibly be affected by light (due to the photosensitivity of the AgCl film, but there are no clear studies to determine magnitudes), and as previously mentioned, a major cause of fluctuation would be due to the presence of gasses in differing amounts in the solution (CO_2_ and N_2_) and to the formation of oxides on the Ag surface during measurement in a non-acidic medium, and to control these factors would require specialized methods not normally employed in most electrochemical laboratories. Nevertheless, despite the above unavoidable circumstances, it was possible to detect an e.m.f. of ~ 1.2 mV (unscaled) with the same polarity without exception whatsoever for all the KCl cell concentrations and this value is well within the range predicted form theory. Attempts at more accurate values (which is not the immediate and main objective of this report) would involve long-term collaboration with specialist laboratories which have the means of imposing the necessary controls on the various variables which affect the e.m.f., including the means of producing the electrode substrate and halide films of unambiguous molecular composition and physical (crystal) orientation. Alternatively, other electrode systems with a much larger predicted cell e.m.f. could be explored, such as the pure metal in equilibrium with its cation, but here the major problem is the definite interference of the oxide layer at the metallic surface which may considerably reduce the second order cell e.m.f. effect.

## 5. Theoretical Models for Cell E.M.F. and Ionic Distributions in AgCl dielectrics. Computations and Results

### 5.1. Computational Method for Cell E.M.F

For cell (1a), the e.m.f. E is given by (2a-b) as:

(48) $$\text{E}={\text{E}}^{\emptyset }(1)-{\text{E}}^{\emptyset }\;(2)+(\text{RT}/\text{F})\text{ln}(1.0)$$

where E^ø^ is the standard potential of the cell concerned, and which is deemed to cancel in practical applications, leading to zero net potential; for what follows, γ_–_ refers to the single ionic activity coefficient which is calculated from the Pitzer equation[37], where for simple 1-1 electrolytes, these equations are symmetrical in the single activity coefficients of both anion and cation and is numerically the same as the mean ionic activity of the salt concerned and may be reduced to the general form:

(49) $$\text{ln}\;\gamma_{-}=\text{F}+2{\text{mB}}_{\text{mx}}+3{\text{C}}_{\text{mx}}{\text{m}}^{2}$$

where F, B_mx_ and C_mx_ are rather complicated functions[37] of the ionic strength I, and m is the (molality) concentration for the species concerned. Since E^ø^ = (−μ(Ag) – μ^o^(Cl^−^) + μ(e) + μ(AgCl))/F and μ_l_ (e) is assumed invariant in μ(e) = μ_F_(e) + μ_l_ (e), (7) becomes by virtue of subtraction at fixed (25 °C) temperature:

(50a) $$\text{E}(\gamma_{-})=\{\mu_{\text{F}},1(\gamma_{-})-\mu_{\text{F},2}(\gamma_{-})\}/\text{F}$$

where the subscripts in μ_F_ refer to the electronic chemical potential of the cell concerned. If A is the total surface area of the electrode then:

(50b) $$\delta \text{N}=\text{A}.\text{Q}(\Delta \Psi_{\text{d}},\text{n})$$

from (3), where δN appears in (4). Since ΔΨ_d_ = ℑ_AgCl|Cl_ − (RT/F)ln m.γ_–_ (where ℑ_AgCl|Cl_ = ℑ^o^), then for any fixed m, ΔΨ_d_ = ΔΨ_d_ (γ_–_). From (3a,b,d), we may write:

(50c) $$\text{Q}={\text{A}}^{\prime }{\text{m}}^{1/2}{\text{F}}_{\text{gen}}[{\text{B}}^{\prime }\Delta \Psi_{\text{d}}(\gamma \_)]$$

F_gen_ is a generic function that is utilized to determine the charge Q on the electrode; the forms utilized here are given in 3(a,b,d), so that for fixed m, Q = Q(γ_–_). Further, we note that at 0.1 N, we wrote:

(50d) $$\Delta \Psi_{\text{d}}={ℑ}^{\text{O}}-\text{RTln}\;({\text{m}}_{2}\text{.}\gamma \_)$$

Since ℑ^o^ = (−μ(Ag) – μ^o^(Cl^−^) + μ(e) + μ(AgCl))/F where ℑ|_neutral_ refers to the neutral state when Q = 0, we write:

(50e) $${ℑ}^{\text{O}}={ℑ|}_{\text{neutral}}+\delta {ℑ}^{\text{O}}$$

where δℑ^o^ = δμ(e)/F is due to the change of ℑ^o^ due to charge transfer. At 0.1 N ℑ^o^ is known precisely (the value 0.394031V is used here for the theoretical calculations), so that Q|_m_ = 0.1 may be calculated, and δℑ^o^ may be determined from (4) and (5). Hence:

(50f) $${ℑ|}_{\text{neutral}}={{ℑ}^{\text{O}}}_{\text{m}=0.1}-\delta {ℑ}^{\text{O}}$$

Since δℑ^o^ = δℑ^o^ (Q) and the functional form δℑ^o^ may be determined, then for any other value of m (other than 0.1 N where N refers to Normality units of concentration), δℑ^o^(mγ) may be computed from (50c). Thus, Q = Q(γ_–_) even if ℑ^o^ is allowed to vary and clearly, μ_F,2_ = μ_F,2_(γ_–_).

The above sub-iterations are used to solve the following equation derived from (48) by the secant method:

(51) $$\text{E}-{\text{E}}^{\emptyset }(1)-{\text{E}}^{\emptyset }(2)=0$$

It will be noted that for each secant iteration in (37), the charge Q must be known, and this information is obtained by solving one of equations (3), depending on electrode choice by a loop utilizing the secant method, and this value of the charge Q is substituted into equations (50) and (51). Equation (45) is solved in exactly the same way irrespective of the type of dielectric model that is used below.

### 5.2. Theoretical Aspects of The Electrode Electrical Double Layer and the AgCl Dielectric

**Figure 7.**
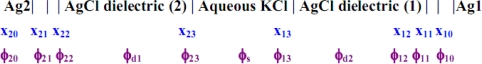
Sketch of interfacial region with associated potentials.

The entire cell may be characterized as having the following potentials for the entire surface perpendicular to the line direction x (from the surface of the metal substrate of the electrode concerned) as depicted in Figure 7.

In Figure 7, x_ij_ represents the interfacial distances of phase boundary j of electrode i, and the ϕ′s are the corresponding potentials at these interfaces; ϕ_s_, ϕ_d1_ and ϕ_d2_ are the bulk solution potentials of the respective regions. Each of the above electrodes i consists of the Ag substrate coated with the AgCl dielectric, which shares a boundary at x_i3_ with the aqueous KCl solution; x_i1,2_ are the inner and outer Helmholtz planes within the dielectric matrix. The model (abbreviated M1) that is favored here and described immediately below focuses on the net electrode reaction:

(52) $$\text{AgCl}(\text{dielectric})\;+\;{\text{e}}^{-}\;(\text{Met}.)\;↔\text{Ag}(\text{Met}.)\;+\;{\text{Cl}}^{-}(\text{in}\;\text{AgCl}\;\text{dielectric})$$

where we envisage the Cl^−^ ions which are freed from the Ag-AgCl interface (x_j0_) to distribute themselves according to the usual Debye-Huckel and Gouy-Chapman assumption of the Boltzmann energy distribution, where Φ, the potential within the dielectric obeys the limit Φ → 0 and dΦ/dx→ 0 as x → ∞. Furthermore, there is overall electroneutrality in (52) because the charge on the Ag metal interface equals the charge of the freed Cl^−^ ions; the actual mechanism of ionic migration of Cl^−^ ions is of no concern here, although it is of central importance in kinetic studies when various defect mechanisms (eg. Frenkel defects) [23,38] are mooted for solid state ionic migrations. In (M1), we do not postulate the free flow of ions from the solution to the AgCl layer treated as a pure dielectric with no strain energy associated with ionic migration, which is the assumption made in for instance the Debye-Huckel theory of ionic activity coefficients. Barring defects, the interstitial distances between fairly rigidly held Ag and Cl atoms is of the order of ~2.77 Å, whilst non-hydrated ionic radii are typically ~1.5Å, which seems to imply that free migration based on strainless movement is not favored and so there is a justified skepticism in utilizing equations (35) and (39) to solve for ionic concentrations within the dielectric in equilibrium with the aqueous salt.

It was discovered that for concentrations from 0.01 – 0.1 M KCl, and ℑ^o^ ~ 0.39 – 0.5 V, the symmetrical neutral electrolyte M^+^Cl^−^ electrode charge equation(3b) and that for the single anion (3c), which describes model M1 and the mechanism in (52) gave (surprisingly) almost the same charge densities σ^m^, i.e.:

(53) $$\begin{array}{l} {\left(2kTɛ{ɛ}_{o}n_{o}\left[\text{exp}\frac{ze}{kT}\left(\Delta \Psi_{d}-\frac{\sigma^{m}x_{2}}{ɛ{ɛ}_{o}}\right)\right]\right)}^{1/2}\\ \approx {\left(8kTɛ{ɛ}_{o}n_{o}\right)}^{1/2}\text{sinh}\left[\left(\frac{ze}{2kT}\right)\left(\Delta \Psi_{d}-\frac{\sigma^{m}x_{2}}{ɛ{ɛ}_{o}}\right)\right] \end{array}$$

for x_d_ ranging from 3.5 to 10^–7^ Å, with a maximum % deviation of ~ 0.8% at high σ^m^ (~ 20,000 μCcm^–2^). Hence these two electrode types are practically indistinguishable for the same single ion concentration *n_o_* and permittivity ε. (The cell e.m.f. would differ by 0.01 mV maximum.) Furthermore, no assumptions involving unknown quantities are required to utilize the single-ion electrode equation (3d), apart from the boundary conditions specified by the Uniqueness Theorem which in this case corresponds to a known physical situation. This is less true for Equations (35) and (39) where the activity coefficients must be known, and where it is assumed that no strain energy accompanies ionic migration from the aqueous to the dielectric phase. In Figure 7 we assume ϕ_i_ = ϕ_s_ = ϕ_i2_. This accords with normal expectation since the average potential at a long distances from a system of dipoles is zero and also with the analysis of Grimley [39] where the potential drop of the solution V_s_(l) to the dielectric given by Λ + V_c_(l) = V_s_(l) (his Figure 1) is negligible, and the net charge per unit area (the actual units used is not specified explicitly) is of the order of 10^–7^ μC (his Figure 2) which is negligible compared to the magnitudes used here (200 – 20 KμCcm^–2^).

**Figure 8.**
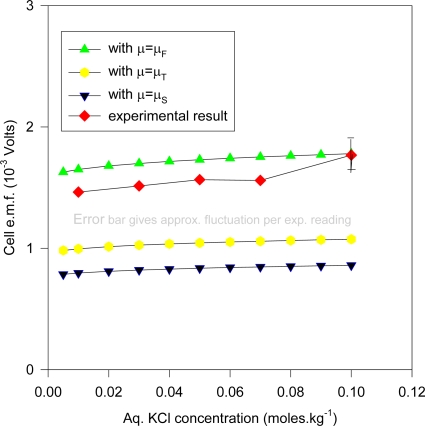
Computed cell e.m.f.s for single Cl^−^ ion electrode for various μ and experimental data.

In order to utilize M1, by the Uniqueness Theorem a boundary condition must be specified. Here we state that within the AgCl dielectric, when x → ∞, the concentration of the Cl^−^ ions must correspond to that at the AgCl-Aqueous interface, implying analytical continuity of the chloride ion concentration; hence we set [Cl^−^] ≡ [Cl^−^]_aq_ with the dielectric constant value of that for AgCl at this interfacial region, and we also set the potential gradient ∂φ/∂**n_i_** = 0 (**n_i_** is the normal unit vector to the interfacial surface). Clearly, the exact concentration and potential profile is very complicated (being a function of defect concentration etc.), but is of no concern here since we are utilizing the Uniqueness Theorem, to uniquely determine the metal charge density if the potential difference and ionic concentration is specified at one surface of the system. The computed e.m.f.’s for cell (1a) for M1 for x_d_ = 0 (corresponding to x_i2_ = x_i1_ = 0) is given in Figure 8 for ε = ε_AgCl_ and for the three chemical potentials mentioned here μ_F_ [Equation (5)], μ_T_ [Equation (7)] and μ_S_ [Equation (16)], together with the experimental result where the e.m.f. values are plotted against the KCl molality. The computations are for sizes of electrodes 2 × 2 cm square, with s2 having thickness 0.0225 mm, whilst s1 has thickness 1mm. It was found that comparable theoretical results (up to 0.01 mV maximum) obtain if s1 varied from 0.5 to 1mm, since at such thicknesses the chemical potentials for s1 would remain fairly constant for the different ionic concentrations.

It will be observed that the experimental results depicted in Figure 8 lie approximately on the curve whose electronic chemical potential is between μ_T_ and μ_F_ (but closer to μ_F_). This result would accord with the low-temperature specific heat ratio γ (Equation (6)) which matches experiment almost exactly in the special case of Ag. It may be reasonably argued that the AgCl dielectric does not play a prominent role (despite its occluding the Ag substrate surface) in determining the cell e.m.f., apart from providing a reservoir for Ag^+^ and Cl^−^ ions.

**Figure 9.**
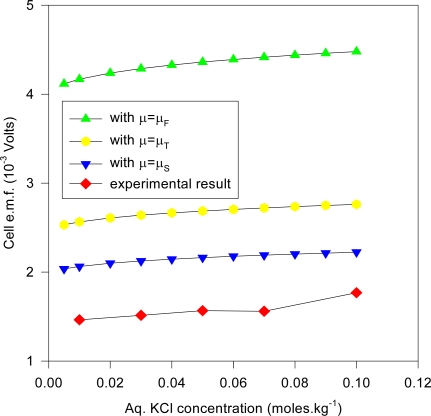
Cell e.m.f. based on H_2_O dielectric constant μ.

It may be reasonably argued that the AgCl dielectric does not play a prominent role (despite its occluding the Ag substrate surface) in determining the cell e.m.f., apart from providing a reservoir for Ag and Cl^−^ ions. This situation corresponds to solving for the cell e.m.f. with the H_2_O dielectric constant (ε = 78.54) at the vicinity of Cl^−^ exchange at the electrode, the results of which are presented in Figure 9 with the same axis variables as for Figure 8 for the three aforementioned chemical potentials.

The types of electrode reactions that are applicable to the results presented in Figure 9 include:

(54) $${\text{K}}^{+}(\text{aq})+\text{AgCl}+{\text{e}}^{-}(\text{Met}.)\to {\text{Cl}}^{-}(\text{aq})+\text{Ag}(\text{Met}.)+{\text{K}}^{+}(\text{aq})$$

with the ions in the aqueous (aq) phase.

It is clear that the computed values are far in excess of the experimental results and so it is safe to infer that AgCl does play a prominent role in distributing the Cl^−^ ions within the dielectric to produce the observed e.m.f. apart from its role as a dielectric ionic reservoir.

It is well known that the Stern modification (GCS systems) with x_d_ ≠ 0 is widely utilized as a refinement to the Gouy-Chapman theory [21,40]. In all these cases, the fundamental assumption is that of thermodynamical equilibrium. Recently, a replica of the current method was applied in a non-equilibrium study[13] where it was concluded that the Stern modification (non-zero x_i2_ of atomic radii dimension in Figure 7) best explained the results of experiments. It is of course questionable to apply equilibrium theoretical models to non-equilibrium situations.

**Figure 10.**
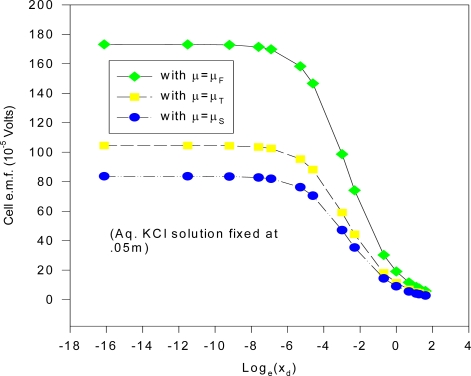
Cell e.m.f. computed for various μ and Helmholtz Inner layer distances X_d_.

The results of using the Stern model for the electrode processes in our equilibrium electrochemical system (1a) with ε = ε_AgCl_, written:

(55) $$\text{AgCl}\;+{\text{e}}^{-}(\text{Ag})\;↔\;{\text{Cl}}^{-}\;(\text{AgCl}\;\text{dielectric})$$

is given in Figure 10 for the chemical potentials as mentioned before, where the cell e.m.f. is plotted against log_e_(x_d_) (where x_d_ = x_i1_), the plane of closest approach in the electrode systems for fixed aqueous KCl ionic concentration (0.05 m) ; x_d_ must correspond to atomic (radius) dimensions (~ 3.5 Å for the hydrated chloride ion).

It is clear from Figure 10 that x_d_ must be much less than 10^–3^ Å, (between 10^–3^ – 10^–7^ Å) for there to be fair agreement with experiment; i.e. x_d_ is far below atomic (or even electron-nuclear atomic core) dimensions, implying that the GC double layer certainly appears more appropriate for our equilibrium study, which incidentally yields good agreement (to at least order of magnitude accuracy) with experimental values for model M1. We can conclude that in those schemes which replicated the present methodology, non-equilibrium effects may well have perturbed the steady-state ionic distribution to such an extent that the GCS equilibrium electrode model appeared to be the most appropriate one, since when we applied this model to a true equilibrium system, the results were not compatible with the experimental results where atomic dimensions are concerned. Although tentative physical considerations might rule out ionic penetrants for the case of the AgCl dielectric, theories have been successfully developed [41,42] to account for ionic penetrants in disordered and other media such as zeolites, with typically large pore sizes of ~ 4.5 Å diameter, where the Debye-Huckel theories apply. There is reason therefore to believe that for electrodes that are coated with such materials [41,42], one may use the methods of Lemma 6 to derive ionic concentration distribution functions for ions in equilibrium between two dielectrics.

In order to illustrate the feasibility of the method (even if it is not very applicable here) we solve Equations (35) and Equations (36) to derive estimated values of the KCl concentration within the AgCl dielectric in equilibrium with the aqueous KCl solution in the case of pure dielectric induced interactions without any lattice strain energy interactions. Unfortunately, little has been developed concerning solid-state ionic activities. As such, we make use of the extended Debye-Huckel expression as the simplest approximation for computing activities γ_±,2_ in the AgCl dielectric (phase 2) relative to parameters from the aqueous phase 1, given by:

(56) $$\begin{array}{l} \gamma_{\pm ,2}=\left(-0.5092{\left(\frac{\rho_{2}}{0.997}\right)}^{1/2}{\left(\frac{{ɛ}_{H_{2}O}}{{ɛ}_{2}}\right)}^{3/2}\sqrt{I}\right)\\ /\left(1+3.29a_{o}\sqrt{I}{\left(\frac{\rho_{2}}{0.997}\right)}^{1/2}{\left(\frac{{ɛ}_{H_{2}O}}{{ɛ}_{2}}\right)}^{1/2}\right) \end{array}$$

**Figure 11.**
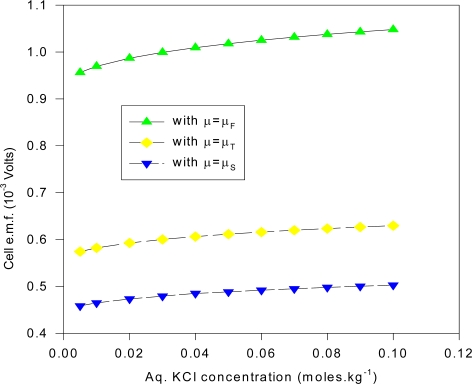
Cell e.m.f. for various μ, based on solution of dielectric equation for KCl concentration in AgCl dielectric.

**Figure 12.**
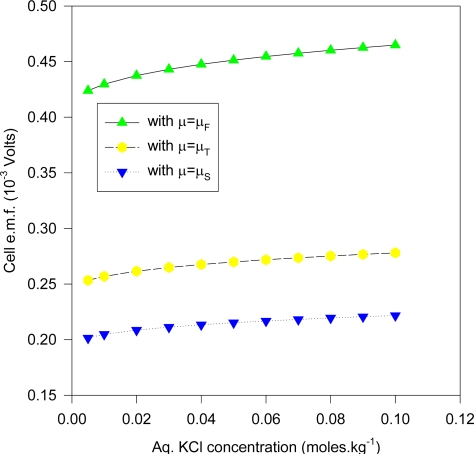
Cell e.m.f. for various μ, based on Born dielectric equation for KCl concentration in AgCl dielectric.

With the above (56) activity coefficient, Equation(35) yields two branches of solution of order ~ 2 × 10^–3^ and ~ 2 × 10^–4^ molal KCl present in the AgCl phase, for aqueous KCl concentration in the range 0.01 – 0.1 m. The average branches from these two branches were taken, because it was noted that by slight adjustment of the ionic radii, a unique solution could be attained by any one fixed concentration of the aqueous KCl. The average solution was monotonically increasing and was linearized to yield the form c = 0.3342 m (c is the KCl concentration in moles L^–1^ in the dielectric layer, m is the aqueous molality). The corresponding values for the ionic radii (Å units) were derived by scaling from Shanon’s crystallographic data [43] and the dimensions for the AgCl unit cell; two values of the radii were used, that for the free aqueous ion (in parenthesis) and the scaled value in the AgCl dielectric, given as 1.6965 (1.81) for Cl^−^, and 1.29357 (1.38) for K^+^. By a process of trial and error, (36) was solved (no bifurcations were observed at low aqueous molalities < 0.05 m) yielding a monotonically increasing function with increasing aqueous concentration, and it was linearized to c = 0.064246 m (again c is the KCl concentration in moles L^–1^ in the dielectric layer, and m is the aqueous molality) ; the ionic radii used was 1.6966 (1.81) for Cl^−^, and 1.3077 (1.38) for K^+^ for the aqueous concentration range 0.01 – 0.1m KCl. We then use these concentrations in the AgCl phase to predict the cell e.m.f. where the bulk ionic concentration corresponds to that in the AgCl dielectric layer, and where the electrode used is either of the kind described by equations (3a) or (3d), corresponding to the electrode equilibria (54) in the dielectric (non-aqueous) phase. The results of the computation are shown in Figures 11 and 12.

Figure 11 plots the cell e.m.f. variation with the aqueous KCl concentration, where the KCl concentration in the dielectric is given by solving (35), whereas Figure 12 is for the same cell system, but with the KCl dielectric concentration derived by solving (36). It is evident that the predicted e.m.f values are somewhat lower than that expected from the experiment, and may be due to the inadequacy of the model, and/or the imprecisely known values of the ionic radii. In cases (such as zeolites) where normal diffusion of ions into the dielectric is a feasible model for ionic migration in electrochemical electrodes, the above results emphasize the need for more precise theories from which accurate ionic radii and activity coefficients may be derived. We note that the results for the Stern modification would show even greater diminution of the predicted cell e.m.f. than those given in Figure 10.

We may conclude from the above discussion that the so-called standard potentials E_i_^ø^ for the various half-cells used to compute the overall cell e.m.f. E with standard E_s_^ø^ written:

(57) $$\text{E}=\{\sum_{\text{i}=1}^{\text{n}}{\text{c}}_{\text{i}}{{\text{E}}_{\text{i}}}^{\emptyset }\}-(\text{RT}/\text{zF})\;\text{ln}\;\text{Q}={{\text{E}}_{\text{S}}}^{\emptyset }-(\text{RT}/\text{zF})\;\text{ln}\;\text{Q}$$

where Q is the overall equilibrium factor [8], and c_i_ the stoichiometric coefficient for each half-cell reaction must be modified for a net z-electron reaction to:

(58) $$\text{E}(\text{m},\gamma )={{\text{E}}_{\text{S}}}^{\emptyset }+\text{δ}{\text{E}}^{\emptyset }(\text{p},\text{m},\gamma )-(\text{RT}/\text{zF})\;\text{ln}\;\text{Q}$$

where δE^ø^ (p,**m,γ**) is the modifying function for the substrate thinfilm or micro-electrode; p refers to the particular arrangement of the electrodes in a cell relative to the standard electrode state where δE^ø^ = 0 and **m** and **γ** are the concentration and activities of the respective electrolyte constituents. The above proposal (including Equation (58)) would account for the “uncertainty in the value of E^o^ ” as reported by Bates and Macaskill in their extensive tabulation and derivation of standard potential values, especially for the Ag/AgCl electrode [44]. They attribute the variation in the reported values of the standard potentials to possible variation in preparative techniques, (and not directly to substrate size which affects the electronic chemical potential), and recommended a standardization procedure which would be abandoned once the “ cases of variability had been identified and eliminated ”. They notice standard e.m.f. differences of up to 0.2 mV amongst different workers; here we observe differences of up to ten times of what the authors quote by exaggerating the dimensions of one of the electrodes by approximately 40 times. The above computations when based on the simple Gouy-Chapman theory seem to yield values closest to experiment; it is noticed that the computational results differ with the choices of the chemical potentials with the Seitz potential registering the greatest difference. Severe discrepancies would possibly arise if one were to connect the Gouy-Chapman theory with the Gibbs adsorption isotherm, which is a standard technique in electrochemical surface analysis [27], where finite size of ions is an extra condition imposed on the equations, leading to the utilization of the GCS model which we have demonstrated to be inadequate. No such attempts are made here to apply such Gibbsian models for 2-dimensional structures to our essentially 3-dimensional models.

Having observed the rough magnitude of the cell e.m.f. which is incompatible with the predictions made on the basis of the GCS interfacial model, we may perhaps attempt to understand why this is so from the point of view of charge accumulation on the electrodes. For the electrode reaction AgCl + e^−^(Met.) ↔ Ag + Cl^−^ (aq) (where we assume that the AgCl dielectric is only a reservoir for ions with no other effect) the charged species in solution includes the cation, Cl^−^ and electrons which are able to percolate through a pathway to the substrate Ag electrode, whereas the Gouy-Chapman (and Stern modification) assumes the ions being the only charge carriers (with finite size for the Stern modification of the Helmholtz double layer). This process pathway still obtains even if we should choose model M1 (but the charges on the electrode would be scaled down by a factor of approximately two, due to the lower value of the dielectric constant for AgCl). For purposes of calculation and comparison, if the (theoretical) substrate-solution potential were fixed at ℑ^o^ = 0.394031 V, then for model M1, the charge per unit area on the electrode may be computed for distances x_2_ = 3.5 Å (GCS model) and x_2_ = 10^–9^ Å (effective GC model). For the For the ordered set of KCl normalities {0.001; 0.005; 0.01; 0.05} with activity coefficients set to unity, the corresponding charge density (μCcm^–2^) for the electrode at x_2_ = 3.5 Å and x_2_ = 10^–9^ Å are the respective ordered sets with values {27.32, 33.46, 36.19, 42.70} and {398.37, 890.77, 1,259.74, 2,816.87}. If it is maintained that at very low concentrations, the Gouy-Chapman model may be accepted as fairly accurate [9,45–47] then at these low concentrations, the integral capacitance is of order ~ 3,000 μCV^–1^cm^–2^, which differs from the Stern model prediction of ~ 100 μCcm^–2^V, which is ~ 30 times less than the value predicted by the simple GC theory at its regime of validity. Hence we can expect from these observations that the Stern theory may not be a suitable description for these types of electrodes, even at low concentrations.

From a strictly theoretical angle, there appears to be a (subtle) and probably minor peculiarity which is assumed in using the GCS model as an ideal polarized electrode. By definition, no charge transfer is envisaged with change of potential, yet there is a constant setting^4^ of σ^m^ = –σ^s^ in order to determine the charge on the electrode (σ^m^) where –σ^s^ = –εε_o_(dϕ/dx)_x = x2_, where ϕ is the solution potential at x_2_ (x_2_ = 0 for the GC model). The reason is attributed to total charge neutrality; if this were the case then one must allow for charge transfer. On the other hand, if there were absolutely no charge transfer then a counter-electrode would have to be placed in the solution for electroneutrality, which would negate the model boundary conditions. As discussed below, this charge neutrality between electrode and solution is accounted for directly in the ideal self-polarizing electrode. These secondary considerations, in addition to those derived from experiment, make us choose the simplified Gouy-Chapman model of the interface because the electrode reaction mentioned above provides an electronic pathway across the interface for charging the electrode; the electronic factor is not considered in the Stern modification, which could lead to a much smaller x_2_ than predicted for an electrode system not reacting with the fluid ions.

For instance, consider the component electrode reactions for the AgCl|Cl electrode:

$$AgCl\;\overset{a^{\prime }}{↔}Ag^{+}(s)+Cl^{-}(s)\;\;and\;\;Ag^{+}(s)\overset{b^{\prime }}{↔}Ag^{+}(m),$$

where on one hand a’ alone would suggest an OHP at distance x_2_ averaged between the hydration radii of Ag^+^(s) and Cl^−^(s). On the other hand, b’ shows that this static OHP cannot exist at x_2_ because of the transfer of charge from the Ag^+^(s) ion to the lattice, implying a smearing of charge between x_2_ and the metal substrate which is precluded in the standard treatment of the OHP layer, which serves as a capacitance layer with no charge distribution between them (when there is no specific adsorption, i.e. x_1_ = 0 in Figure 1). Thus, it would appear that the interfacial region would resemble more to the simple GC model with x_2_ = 0; also, the σ^m^ = –σ equation would obtain here without a need for a counterbalancing electrode since it is assumed that the charges separate in thermodynamical equilibrium from a neutral (electrode plus solution) combination, i.e. σ^m^+σ^s^ = 0 at all times up to equilibrium. Lastly, according to Sparnaay [45–47] the corrections due to finite ion size and the excluded volume tend to cancel in some systems, where it is stated [47]: “It is now generally accepted [48] (that the corrections tend to cancel and that the original Poisson-Boltzmann equation leads to a fairly (*eg.* within 20% of the uncorrected potential value) reliable distribution law of the ions near a charged wall.” We postulate that Sparnaay’s observation may well be due to the above smearing-out effect of the charge which would obliterate a static OHP surface. The following theoretical form is proposed for extensions of the Gouy model; since the relative permittivity ε(x) can be calculated from ionic concentrations which vary from the distance x of the electrode surface, we integrate the following Gauss expression from the surface of the electrode to infinity as follows. Since the field strength ξ = dϕ/dx, where ϕ is the potential function, the charge –Q on unit area of the planar electrode is:

(59) $$Q={ɛ}_{o}\int_{a}^{\infty }ɛ(x)(\nabla .\xi )\;\;dx$$

where a > 0 for the Stern - type modification, where a is the closest approach distance to the electrode surface, and ε_o_ is the permittivity of free space. Further modifications to this would follow along similar lines as for previous discussions on this topic such as excluded volume effects and the finite size of ions, but it is anticipated that the ε(x) term would partially account for some of these factors.

## 6. Conclusions

Most of the theorems presented here may be corroborated by well established experimental data. The preliminary experiment presented here indicate that electrode size effects which affect the cell e.m.f. does exist, and despite the 20% uncertainty in the measurement, due mainly to the unavoidable variation of physical properties of the substrate silver metal and the silver chloride constituents, are of a magnitude which does not contradict the standard theories, such as the postulated electronic chemical potential of Ag inferred from data such as its low temperature specific heat. Where change of e.m.f. with size electrode size is concerned, the problem then becomes one of determining the precise magnitude of these differences with the change of the thermodynamical variables (including size), so that a clear electrochemical standard may be prescribed. From a practical point of view, the standardization of electrodes following the recommendation of people like Bates and Macaskill [44] seems inevitable, but with the proviso that size and other variables which affect the electronic chemical potentials be included in the standardization specification.

The preceding theoretical results concerning the e.m.f. differences may conceivably be quantitatively improved further by using modified theories of the electrode-solution potential[49] and charge distributions together with Fermi statistics taking into account Brilloun zones where the application of the statistical mechanics used here is not suitable (eg. for Pt and the Transition elements). It is possible to verify some of the above conclusions by say creating single element electrodes where the above statistical mechanics is applicable (such as Ag, with net reaction Ag^+^(aq) + e^−^ ↔ Ag (solid)) of different - up to thinfilm - dimensions to attempt to observe possible size effects on the cell e.m.f. (if the oxide-layer and Ag structural problems can be overcome!). Then, an empirically based set of Tables may be constructed for all such electrodes if for the purposes at hand the changes of potential are considered significant since at present exact calculations on the (Fermi) electron distribution for many metallic elements only seem to be tentative and semi-quantitative and are also based on single-crystal assumptions, none of which are strictly valid for real electrodes. We also expect the effects to vary depending on the nature of the Fermi surface; some metals like Ag are predicted to have discernable effects on the standard potential with size of the electrode, whereas others may show less variation of size effects mentioned above due to the effects of the zone boundaries partitioning the electronic states.

Recently, it has been shown [50] that there is a rather “puzzling” small difference between rate constants of standard size and ultramicroelectrodes for Pt, and these differences may well be due to electronic perturbations due to size of E° and other physical properties as mentioned in the text. However, the current methodologies [51] of examining redox potentials and E° do not appear to consider size effects. We note that the theoretical computations were carried out in relation to preliminary experimental measurements where the thinnest electrode of Ag substrate was 0.025 mm for instance and the results in Figure 6 and elsewhere refer to such a system which was experimentally fabricated. To test for smaller thicknesses would involve fabrication of thin film systems requiring very sophisticated techniques to be found in specialist laboratories, and so hypothetical calculations were not conducted, but this is one further area for future research. However, as the electrodes become thinner, so that the charge density builds up within and not just the surface, there is a point at which its electrical capacitance ceases to be linear, and where the free-electron model cannot be used to compute the chemical potential and the electrical potential since the actual charge transferred cannot be related to the proposed model. If the substrate of the electrode is negatively charged for instance, there will come of point where further electrons cannot be absorbed onto the surface and volume regions. Another area therefore of future study could involve a DFT (density functional theory) study of charge transfer and calculation of the potentials at which there is a breakdown of the retention of charge beyond the electrode capacitance.

One of the consequences of the electrode theorems above, corroborated by the experimental results is that eq. (31) and its analogues apply where the capacitance of the electrode is concerned since the GC interface has empirically been shown to be the more appropriate model. Furthermore, for the concentration range 0.01 – 0.10 M, the theoretical calculations show δℑ ~ 10^–4^ V, i.e. ℑ^o^ is approximately constant. If C_d_ is determined from capacitance studies, then the elusive single–ion activity coefficient may be determined exactly if either ℑ^o^ is known, or is assumed constant, as indicated here.

The other lemmas, such as those concerning the self-polarizing electrode are readily applied to a variety of problems such as those concerning capacitance; in particular, these electrodes also suggest, by way of operation another 3-dimensional treatment of the interfaces, where at present the 2-dimensional utilization of the Gibbs surface excess and surface tension equations are the main descriptions.

Concerning the electronic chemical potential, if accurate cell e.m.f. measurements are possible, then the (solid state) electronic correlation energy ε_xc_(n) may be determined by using the expression such as μ_T_ [Equation (17)] ], and the constants found in Wigner’s interpolative expression (derived from ab initio quantum mechanical calculation) may be modified on a case-by case basis for some metals; since the low resolution experiment mentioned here and other more accurate and established experiments (such as the low temperature specific heat of Ag) seem to indicate that the free electrons of Ag metal behave thermodynamically to a high degree of accuracy as a highly degenerate electron gas, it would be rash for solid state physicists to automatically apply, without recourse to a case-by-case examination, the quantum-mechanically calculated correlation energy of the electron gas ε_xc_ (which is generally a function of the free electron density only, at fixed temperature, and pressure) to problems, since for Ag, its effective correlation energy may be taken to be zero where some thermodynamical properties are concerned, where, on the other hand, ε_xc_ yields a non-zero value.

## Epilogue

The above work shows that with even very elementary methods, and relatively non-specialist skills, one can still pose and solve problems in a qualitative sense that suggests quantitative directions, and within a context that might appear attractive to those fragile political, social and scientific environments. But such solutions are not possible without in the first place the existence of a *style* of doing research that is founded on elementary considerations that are independently conceived that can invite further investigation and participation in an open manner. The only achievement of this piece of work was that it was carried out in a manner that made such possibilities available to all, especially those not emplaced in a secure research grid, which is the fate of the majority by only concentrating on fundamental and elementary issues. Concerning basics, we did not immediately resort to the techniques pioneered by specialists (e.g. using the hypernetted chain equations approximations -HNC- and concepts of surfaces to model electrode processes and solution interactions, and the DFT methods available in the late 1990’s) [52], but rather viewed the subject in broad and general terms that nevertheless could question some of the most fundamental assumptions and suggest new interpretations. More specifically, we showed from elementary considerations that the Stern layer did not account for electron interchange with the electrode and solution [Sec. 12.3.3] [9]; further that conventional charge calculations did not come from solving non-linear equations but the charge densities were derived via the capacitance ansatz [Equation 12.3.29] [9] which amounts to an assumption of the truth of the capacitance equation. The computations show (paragraph between Equation 58 and Equation 59 of this review ]) a discrepancy of the order of magnitude 300 for a typical outer layer solvated ionic radius of 3.5 Å. Hence the experimental results that are routinely reported are based on the Capacitance ansatz with no counter-check by solving numerically for the charge density quantities. A possible reason why this method endures [53–55] is that qualitatively, it depicts the type of behavior expected from the Stern model [Figure 12.3.7, p. 511] [9] and [Figure 5.5, p. 157, Sec. 5.1] [56] but without referring to the charge quantities as derived from basic calculation [13]. In the original paper written a decade ago, a simple extrapolative formula was used to calculate the chemical potential change without assuming the Faraday result of the accumulation of charge at the surface only which might be seen as a means of minimizing the free energy of the system; specialists have assumed constant chemical potential in the bulk of the lattice and assumed “infinite” lattices and canceling image charges to compute surface electronic properties [57] but the assumptions used in these methods may not be very correct, according to some other interpretations [58]. The charge build up at the surface is a net and not instantaneous experimental result as deduced by Faraday in his very elegant ice pail and other macroscopic experiments where care must be exercised in extrapolation to smaller size and dimensions, and also different geometries. One could envisage at the first instance excess electrons uniformly placed in a lattice matrix; at first one might assume from classical reasoning that there exists a net or effective work function potential preventing electrons from leaving the body with its finite surface area, and that the excess electrons in the lattice would repel each other and migrate to the surface, but the force of repulsion that electrons experience normal to the surface vector – the tangential surface- might imply that there might be a more complex distribution of charge that does allow for full discharge of excess charge through the surface but where there might exist instantaneous configurations of excess charge well below the surface region. The Faradaic assumption is also used for more sophisticated calculations of surface charge properties based on “infinite” lattice [59] boundary conditions. It is my opinion that it might be more logical to consider systems as finite entities, in keeping with physical reality and to devise periodic boundary conditions over these finite dimensions. The point here, however, is that the problem of calculating the actual chemical potential of electrodes is an open issue amongst specialists: here we used very elementary “back-of-the-envelop” elementary reasoning of a qualitative kind to suggest that the electrochemical assumptions are just that and nothing more and that these assumptions are not supported by the very elementary calculations performed here assuming a uniform electron gas for the Ag lattice of excess charge with no surface effects relative to the other assumptions concerning the non-exchange energies.

## Appendix

The tables below represent the raw numerical values that were used in the quantitative Figures found in the main text. They are provided here merely to aid in quantitative analysis of the plots. The dimensions and scales of these numbers can be inferred from the corresponding Figures and plots in the main text and so column captions are superfluous.

**Table 1.** Numbers used for plotting Figure 3 for variation of ϕ_2_ with ionic concentration.

−3.0	0.2018	−3	−0.2188	−3.0	−0.1469	−3.0	0.1689
0.0	0.068	0.0	−0.0719	−0.0	−0.0125	0.0	0.0347

**Table 2.** Numbers used for plotting Figure 4 for charge on Hg electrode in contact with 1M electrolytes.

−0.9536	−24.0	−1.0150	−20.0	−1.0731	−20.0	−1.1077	−20.0
−0.5076	−16.0	−0.7385	−16.0	−0.8308	−16.0	−0.8539	−16.0
−0.3115	−12.0	−0.5192	−12.0	−0.6000	−12.0	−0.6115	−12.0
−0.1615	−8.0	−0.3000	−8.0	−0.3577	−8.0	−0.3692	−8.0
0.0000	0.0	0.0000	0.0	0.0000	0.0	0.0000	0.0
0.1269	8.0	0.3000	8.0	0.2539	8.0	0.2539	8.0
0.2054	12.0	0.4385	12.0	0.3462	12.0	0.3000	12.0
0.2308	16.0	0.6000	16.0	0.4270	16.0	0.3577	16.0

**Table 3.** Numbers used for plotting Figure 6 for plot of e.m.f. vs. surface area for electrode s2.

0.05	−0.2414	−0.1438	−0.1146
1.00	1.9329	1.1536	0.9194
2.00	4.2022	2.5123	2.0033
4.00	8.6818	5.2084	4.1575
6.00	13.0853	7.8765	6.2939
8.00	17.4150	10.5176	8.4127
9.00	19.5543	11.8281	9.4656

**Table 4.** Numbers used for plotting Figure 8 for experimental results vs. single electrode e.m.f.s, with ε_AgCl_.

5.0000^−3^	1.6284	0.9826	0.7858	xexp	yexp
0.0100	1.6501	0.9959	0.7965	0.0100	1.4640
0.0200	1.6790	1.0135	0.8107	0.0300	1.5140
0.0300	1.6996	1.0263	0.8208	0.0500	1.5660
0.0400	1.7162	1.0364	0.8290	0.0700	1.5600
0.0500	1.7302	1.0450	0.8358	0.1000	1.7680
0.0600	1.7423	1.0524	0.8418		
0.0700	1.7531	1.0589	0.8471		
0.0800	1.7627	1.0649	0.8519		
0.0900	1.7716	1.0703	0.8562		
0.1000	1.7797	1.0752	0.8602		

**Table 5.** Numbers used for plotting Figure 9 for experimental results vs. single electrode e.m.f.s, with ε_H2O_.

5.0000e-3	4.1163	2.5309	2.0358	xexpt	yexpt
0.0100	4.1686	2.5640	2.0628	0.0100	1.4640
0.0200	4.2380	2.6084	2.0985	0.0300	1.5140
0.0400	4.2877	2.6396	2.1242	0.0500	1.5660
0.0500	4.3275	2.6649	2.1447	0.0700	1.5600
0.0600	4.3610	2.6861	2.1620	0.1000	1.7680
0.0700	4.3899	2.7046	2.1771		
0.0800	4.4156	2.7210	2.1904		
0.0900	4.4387	2.7357	2.2023		
0.1000	4.4598	2.7491	2.2133		
0.0300	4.4791	2.7614	2.2238		

**Table 6.** Numbers used for plotting Figure 10 for GCS e.m.f. prediction with ε_AgCl_.

−20.0223	173.1564	104.5700	83.6511
−15.4172	173.1228	104.558	83.6343
−13.1146	172.8186	104.3690	83.4821
−11.5052	171.4856	103.5420	82.8155
−10.8120	169.8613	102.5360	82.0041
−9.2026	158.3099	95.4024	76.2588
−8.5095	146.6338	88.2300	70.4922
−6.9000	98.6750	59.0810	47.1330
−6.2068	74.1631	44.3246	35.3413
−4.5974	30.2894	18.0615	14.3911
−3.9043	19.1034	11.3867	9.0717
−3.2112	11.6658	6.9520	5.5382
−2.8057	8.6350	5.1459	4.0993
−2.6515	7.6868	4.5803	3.6487
−2.2949	5.8486	3.4848	2.7760

**Table 7.** Numbers used for plotting Figure 11 for e.m.f. of cell from solving the dielectric equation.

5.0000e-3	0.9565	0.5742	0.4584
0.0100	0.9696	0.5821	0.4648
0.0200	0.9869	0.5926	0.4732
0.0300	0.9993	0.6001	0.4792
0.0400	1.0093	0.6062	0.4848
0.0500	1.0178	0.6113	0.4881
0.0600	1.0252	0.6158	0.4917
0.0700	1.0317	0.6197	0.4949
0.0800	1.0376	0.6233	0.4977
0.0900	1.0430	0.6266	0.5003
0.1000	1.0479	0.6296	0.5028

**Table 8.** Numbers used for plotting Figure 12 for e.m.f. of cell predicted by solving the Born dielectric equation.

5.0000e-3	0.4239	0.2534	0.2015
0.0100	0.4297	0.2569	0.2048
0.0200	0.4375	0.2615	0.2086
0.0300	0.4430	0.2649	0.2112
0.0400	0.4475	0.2676	0.2134
0.0500	0.4513	0.2699	0.2152
0.0600	0.4546	0.2718	0.2168
0.0700	0.4575	0.2736	0.2182
0.0800	0.4602	0.2752	0.2194
0.0900	0.4626	0.2766	0.2206
0.1000	0.4648	0.2780	0.2217

Miscellaneous: For theoretical calculation, the exposed area of s1 corresponded to 6.8926 cm^2^ for the penetration depth of 1.920 cm. All computations were in double precision (32-bit machine).
